# Inheritance of DNA Transferred from American Trypanosomes to Human Hosts

**DOI:** 10.1371/journal.pone.0009181

**Published:** 2010-02-12

**Authors:** Mariana M. Hecht, Nadjar Nitz, Perla F. Araujo, Alessandro O. Sousa, Ana de Cássia Rosa, Dawidson A. Gomes, Eduardo Leonardecz, Antonio R. L. Teixeira

**Affiliations:** 1 Chagas Disease Multidisciplinary Research Laboratory, Faculty of Medicine, University of Brasilia, Brasília, Federal District, Brazil; 2 Department of Biochemistry and Immunology, Institute of Biological Sciences, Federal University of Minas Gerais, Belo Horizonte, Minas Gerais, Brazil; Smithsonian Institution National Zoological Park, United States of America

## Abstract

Interspecies DNA transfer is a major biological process leading to the accumulation of mutations inherited by sexual reproduction among eukaryotes. Lateral DNA transfer events and their inheritance has been challenging to document. In this study we modified a thermal asymmetric interlaced PCR by using additional targeted primers, along with Southern blots, fluorescence techniques, and bioinformatics, to identify lateral DNA transfer events from parasite to host. Instances of naturally occurring human infections by *Trypanosoma cruzi* are documented, where mitochondrial minicircles integrated mainly into retrotransposable LINE-1 of various chromosomes. The founders of five families show minicircle integrations that were transferred vertically to their progeny. Microhomology end-joining of 6 to 22 AC-rich nucleotide repeats in the minicircles and host DNA mediates foreign DNA integration. Heterogeneous minicircle sequences were distributed randomly among families, with diversity increasing due to subsequent rearrangement of inserted fragments. Mosaic recombination and hitchhiking on retrotransposition events to different loci were more prevalent in germ line as compared to somatic cells. Potential new genes, pseudogenes, and knockouts were identified. A pathway of minicircle integration and maintenance in the host genome is suggested. Thus, infection by *T. cruzi* has the unexpected consequence of increasing human genetic diversity, and Chagas disease may be a fortuitous share of negative selection. This demonstration of contemporary transfer of eukaryotic DNA to the human genome and its subsequent inheritance by descendants introduces a significant change in the scientific concept of evolutionary biology and medicine.

## Introduction

*Trypanosoma cruzi* infections (American trypanosomiasis) affect several million people in Latin America [1], usually in the absence of signs and symptoms of an acute illness [2]. The population carrying *T. cruzi* infections are recognized during epidemiological surveys or routine medical examinations [3]. However, less than one third of all the *T. cruzi*-infected individuals will present symptoms of Chagas disease, usually when they are over 30 years of age. The disease manifests in the heart in 94.5% of cases. The remaining 5.5% affects the esophagus (megaesophagus) or the colon (megacolon). The variable clinical manifestations of Chagas disease are related to disabilities that may accompany a Chagas patient for his entire life [4].

Experimental *T. cruzi* infections in rabbits are characterized by lack of morbidity and mortality in the acute phase when the parasitemia is high, but the animals frequently die from chronic Chagas heart disease with a lack of demonstrable parasitemia [5], [6]. The treatment of Chagas disease patients [7] and of acutely *T. cruzi*-infected rabbits [8] with anti-trypanosomal nitro derivatives curtailed parasitemia, but treated patients and rabbits died of chronic Chagas heart disease. Furthermore, histopathological examination of heart tissue showed the typical myocarditis of Chagas disease, whereby mononuclear cells of the immune system present in inflammatory infiltrates lyse and destroy heart fibers without the parasite in physical proximity [3]. What could be sustaining the destructive lesions in the heart of nitro derivative-treated Chagas patients and rabbits? In order to answer this question we hypothesized that the parasite DNA could be retained in the body and that the resulting genomic alterations could explain the rejection of heart tissue by the host immune system. Thus, we postulated lateral transfer of parasite DNA to the host genome [9], [10].

The protozoa (Eukaryota, Excavata, Euglenozoa) of the Order Kinetoplastida include *T. cruzi*, a parasite of medical and veterinary importance. The enzootic infections affect over 1250 wild mammal species dwelling in the America between parallels 42°N in the United States and 42°S in Argentina. Infections in humans occur where people live in close proximity to blood-sucking insect (Hemiptera; Triatominae) transmitters of *T. cruzi* [3], [11].

An impressive feature of virulent *T. cruzi* is the high amount of extra-nuclear mitochondrial DNA known as the kinetoplast (kDNA), accounting for 15 to 30% of the total cellular DNA, with a massive number of minicircles in a catenated network [12], [13]. The kDNA is comprised of approximately 15 thousand 1.4-kb minicircles [13]–[16]. Each minicircle consists of four constant regions interspersed by variable sequences that contain guide RNA genes required for uridine insertion/deletion RNA editing [17]. Minicircle constant sequence blocks (CSB) are specific sites for replication, transcription, and recombination [18], as well as points for lateral transfer of kDNA sequences to the host cell nucleus [19], [20].

Lateral DNA transfer (LDT) is both the process and the successful outcome of the transfer of genetic material from one organism to another. A current paradigm change in evolutionary biology addresses the important role of LDT and its vertical inheritance in the cumbersome representation of our evolutionary history [21]. The misbehavior of phylogenetic trees has been the subject of debate for the past two decades [22]–[24]. A possible explanation is the extensive occurrence of LDT such that no tree of life will ever be possible [21]. A burgeoning opposition has argued against the adaptive importance of LDT in view of the inadequacy of techniques to detect transfer between closely related organisms [25]–[27]. Thus, unless additional reliable evidence is presented, the idea of ubiquitous LDT may be invoked only as a last resource [28]–[29].

Evolutionary forces driving organismal complexity at the molecular level modulate genotypic and phenotypic diversity, but their relative importance in population genetics is still debatable [30]–[34]. In this respect, the forces increasing genetic variation and phenotypic complexity could be influenced by the transposable elements present in eukaryotes [35]–[37]. The retroposon long interspersed nuclear element-1 (LINE-1) makes up to 17% of the human genome [36]. LINEs are approximately 6 kb in length and have a 5′-untranslated region (UTR), two open reading frames (ORFs), and a polyadenylated 3′ UTR [38], [39]. LINEs have structural similarities with non-autonomous short interspersed nuclear elements (SINEs) that share topological microhomologies within the host genome [40], [41]. Autonomous mobilization of LINEs can create new genes, pseudogenes, and gene loss, alter gene expression [38]–[41], and is associated with disease [42]–[44].

Discussions about the influence of transposable elements in increasing levels of genetic variation are limited by the effectiveness of our analytical tools [30]. For example, the history of LDTs has been accelarated by the advent of high throughput genome sequencing [25], [45]. The shortage of information about contemporary DNA transfer among eukaryotes stems from the randomness of integration and lack of information about foreign and target DNA topology, making LDT detection inaccessible to an effective methodological approach. Perhaps a crucial asset for detection of ongoing DNA transfer and its vertical inheritance is the choice of an intracellular pathogen persisting in the host throughout its lifetime, providing the epidemiological requirements for a LDT population genetics study. This is the case of cryptic *T. cruzi* infection and endemic Chagas disease [2], [3]. *T. cruzi* resistance to elimination by macrophages [46] and its encryption inside non-phagocyte muscle cells [47] with occasional release of parasitic forms into the bloodstream may be fundamental for LDT. The choice of Chagas disease as a model to study active LDT is further validated by important host-parasite intrinsic features, such as: 1) *T. cruzi* intracellular forms leave the parasitophorous vacuole of the host cell and remain free to divide in the cytoplasm [48], [49]; 2) during cell division minicircles are cleaved by topoisomerases [50] and could migrate to the host cell nucleus [51], [52]; 3) and, *T. cruzi* minicircles, with four adenine- and cytosine-rich (AC-rich) CSBs [18], [19], can mediate kDNA insertion into the host genome. All these factors may influence lateral kDNA transfer (LkDT) from *T. cruzi* to host cells [9], [10], [20], [53]. Our studies were stimulated by the demonstration of existing LkDT events in close proximity as disclosed by PCR using opposing kDNA primers where minicircle fragments had integrated into the host LINE-1 elements [20].

The immediate recognition of other LkDT events sparsely distributed throughout the genome was limited by unsuitable PCR-based techniques. Inverse PCR [54], [55], adapter-ligation-mediated PCR [56]–[58], or rapid amplifying cDNA ends (5′RACE) [59]–[60] frequently fail to penetrate into the host flanking DNA. Thermal asymmetric interlaced PCR (TAIL-PCR) is a nested PCR used for amplification of unknown template DNA fragments [61]–[64]. The reaction includes three nested specific primers in consecutive reactions combined with arbitrary degenerate primers used at a low melting temperature. As the amplification of DNA products are controlled by temperature, low-stringency cycles create sites for primer annealing, while high-stringency cycles augment the specificity of the nested amplification products [62]. Regardless of the improvements made, identification of integrated minicircles sequences using arbitrary degenerate primers continued to be a difficult task. To solve the problem we replaced the degenerate primers with LINE-1-specific primers from the conserved 5′-UTR, open reading frame 2 (ORF2), and from the 3′-UTR region. These primers were used successfully in combination with nested kDNA primer sets in a targeted primer TAIL-PCR (*tp*TAIL-PCR).

Here we document the presence of parasite DNA in the genomes of Chagas patients and their descendants. Five families were chosen on the basis of the founders having Chagas disease as confirmed by specific anti-*T. cruzi* antibodies and/or nuclear DNA (nDNA) signatures. The *tp*TAIL-PCR technique was used to demonstrate the rate of LDT and to understand the consequences of LkDT in the families studied. The *tp*TAIL-PCR based on kDNA and LINE-1 sequences showed that *T. cruzi* minicircles integrate primarily into host genome transposable elements. Furthermore, the integrated minicircle fragments were inherited by Chagas disease patient progeny. Minicircle integrations into nearly all human chromosomes are characterized. We demonstrate kDNA minicircle sequences concentrated within LINEs, and multiple integration events mobilizing, and thus ‘hitchhiking’, to other chromosomes, resulting in disruption of coding regions and gene loss.

## Methods

### Population Studied and Diagnostics of the *Trypanosoma cruzi* Infections

**Figure 1:**
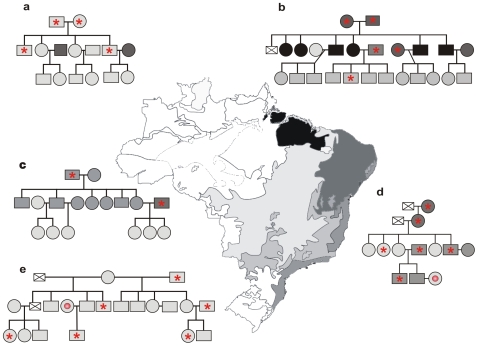
Origin of family members in this study. Map showing five main ecosystems in Brazil: light gray, Cerrado, savannah-like; intermediate gray, Inland Atlantic forest; gray, Coastal Atlantic forest; dark gray, Caatinga, dry shrubs; black, Amazon-Tocantins rain-forest. Family pedigrees **a-**to**-e** showing intermingling of human populations from the five main ecosystems. Based on their epidemiological histories, families **a**, **b**, and **c** live in the urban Federal District of Brazil, where individuals are not exposed to the insect transmitters of *T. cruzi* infection. By contrast, country families **d** and **e** live in houses infested with the cone-nosed kissing bugs, vectors of *T. cruzi* infection. The red asterisks indicate family members with chronic *T. cruzi* infection as detected by ELISA, hemagglutination, and immunofluorescence exams, and by the parasite DNA signature. The red disks indicate individuals showing the parasite DNA signature in absence of anti-*T. cruzi* antibodies.

The Ethical Committee on Human Research of the Brasilia University Medical School approved the research protocol, and all procedures were carried out in compliance with Brazilian regulations and international guidelines. Each volunteer signed an informed consent form. Among the 87 volunteers there were 84 family members over 5 and under 70 years of age (**Figure 1**). The founders of families **a**, **b**, and **c** were patients enrolled in a 20-year program for health assistance to chagasic patients in the Federal District, Brazil, where they were free from triatomine exposure for the last three decades. The founders and progeny of families **d** and **e** lived in the county of Bomfinópolis, Minas Gerais State, Brazil, where they had been in contact with triatomine transmitters of *T. cruzi*. A control group included Chagas-free individuals (2 males and 3 females) born on the European Continent, who were never in contact with triatomines. Patients 1, 2, and 3 were used as positive controls because they had parasitological demonstration of *T. cruzi* in venous blood grown in blood-agar slants with an overlay of liver infusion tryptose medium. The remaining Chagas patient infections were identified by enzyme-linked immunosorbent assay (ELISA), hemagglutination and immunofluorescence assays, which showed specific anti-*T. cruzi* antibodies in serum samples, and/or by the parasite nDNA signature [7].

### Macrophage and Parasite Growth

Human macrophage line U937 (ATCC number: CRL-1593.2) and *T. cruzi* were each grown as described [20]. Epimastigote forms of *T. cruzi* were grown in liver-infusion tryptose axenic medium at 27°C. *Leishmania (viannia) braziliensis* was grown in Dulbecco’s modified Eagle’s medium (DMEM) with 20% fetal bovine serum. The parasites were harvested in exponential growth phase.

### Obtaining Samples for DNA Extraction

DNA samples were extracted from U937 macrophages, *T. cruzi* epimastigotes, and *L. braziliensis* promastigotes [20]. Venous blood (20 mL) was drawn and mononuclear cells separated using a Ficoll-Paque (GE Healthcare, São Paulo, Brazil) gradient. The genomic DNA from blood samples was preformatted as described by Sambrook and Russel [65]. Males above 18 and below 65 years of age collected their sperm, which was suspended immediately in DMEM (1∶4 v/v), pH 7.4 (Sigma-Aldrich, São Paulo, Brazil). The sperm cells were allowed to rest for 45 min at 5% CO_2_, 37°C; free-swimming spermatozoa were recovered from the supernatant, centrifuged at 13000 x *g* for 5 min, and transferred to extraction buffer (10 mM Tris-HCL, pH 8.0, containing 10 mM NaCl, 20 mM EDTA, 1% SDS, 0.04% proteinase-K, and 1% DTT) [66].

### Nucleic Acid Analyses

DNA was extracted from peripheral blood mononuclear cells collected from three patients with parasitological demonstration of the chronic *T. cruzi* infections, from 84 individuals in five study families, from five European volunteers used as controls, and from U937 macrophages. Mitochondrial kDNA was obtained from *T. cruzi* epimastigote forms [20]. The standard PCR procedure [7], [67], [68] was used to demonstrate kDNA and nDNA signatures in test samples DNA. The PCRs were carried out using template DNA that was 20-fold above the levels of detection, so as to increase sensitivity [69], [70]. Each pair of primers (10 µM) was used with 2 U *Taq* and 0.2 mM dNTP, and 1.5 mM MgCl_2_ in a 25-µL final volume. In control experiments each PCR was conducted with a template DNA-free blank, DNA from control individuals born in the European Continent, DNA from a Chagas patient with parasitological demonstration of *T. cruzi* infection, and parasite DNA.

Southern hybridizations were performed with *Eco*RI digestions of test patient DNA. The rational for choice of *Eco*RI to digest genomic DNA was the presence of single cuts in the kDNA minicircle and in the LINE-1 consensus sequences (**Figure S1**). The digestions of DNA from test patients, uninfected controls, and *T. cruzi* cells were subjected to electrophoresis in 0.8% agarose gels at 50 V overnight at 4°C. Hybridizations with a radioactively labeled kDNA probe were performed as recommended by standard Southern blot protocols [65]. The membrane was washed twice for 15 min with 2X SSC and 0.1% SDS, twice for 15 min each with 0.2X SSC and 0.1% SDS, and autoradiographed for variable periods of time.

### Primers and Probes Used in PCR and Southern Analysis

The primer sets used were S35/36 and S34/67 for kDNA [67], and Tcz1/2 for nDNA [68]. PCR products were transferred to a positively charged nylon membrane by the alkaline method. DNA sequence probes labeled with [α-^32^P] dATP following the protocol of the Random Primer Labeling Kit (Invitrogen, Carlsbad, CA) were used for: 1) total *T. cruzi* kDNA (∼1.4 kb); 2) kDNA constant region (kCR) probe TTTTGGTTTTGGGAGGGGCGTTCAAATTTTGGCCCGAAAATTCATGCATCTCCCCCGTACATTATTTGGCCGAAAATGGGGGTTCGATGGAGGTGAGGTTCGATTGGGGTTGGTGT-3′) representing a constant region of *T. cruzi* kDNA minicircle (GenBank accession number AF399841), with primers S67 and S34 underlined, respectively, at the extremities, and the nested S35 primer from 65 to 46 nts; 3) the 350-bp probe resulting from *Nsi*I digest of kDNA; 4) the 198-bp fragment obtained with the Tcz1/2 primers; and, 5) the LINE-1 clone 198 probe shown in Figure S1. Further PCR examinations were performed aimed at validation of amplifications of kDNA integration sites in the DNA of somatic as well as germ-line cells. These experiments were performed with 100 ng test DNA, 2 mM MgCl_2_, 10 µM each of kDNA S67 antisense and LINE L1-5 primer, 0.2 mM dNTPs and 2.5 U *Taq* Platinum. The program used was: 95°C for 5 min followed by 35 cycles at 95°C for 30 secs, 64°C for 1 min, and 72°C for 3 min; the reaction was maintained at 72°C for 5 min, and stored thereafter at 4°C.

### *tpTAIL-*PCR, Validation of the *tpTAIL-*PCR, Cloning and Sequencing

Thermal asymmetric interlaced (TAIL)-PCR [63], [64], which uses degenerate primers to amplify exogenous DNA integrations, did not yield kDNA linked to retrotransposon flanking sequences (data not shown). To improve the TAIL-PCR we substituted the degenerate primers with LINE-1 targeting primers (*tp*), which were used in combination with kDNA primers in a nested *tp*Tail PCR. This procedure does not involve restriction enzyme digestions and requires no manipulation of genomic DNA before amplification.

In the first round of amplifications, each reaction used 200 ng of DNA template, 2.5 mM MgCl_2_, 10 µM kDNA primer (S34 or S67), 0.2 mM dNTPs, 2.5 U *Taq* Platinum (Invitrogen, Carlsbad, CA). The kDNA primers were used in combination with 1 µM of each individual L1 primer (L1-1 to L1-6; **Table S1**). The *tp* annealing temperatures ranged from 57.9 to 60.1°C for kDNA primers, and from 59.9 to 65.6°C for LINE-1 primers. These temperatures are much higher than those (circa 45°C) required for the arbitrary degenerate primers in TAIL-PCR. The temperatures and cycles used (MyCycle Thermo Cycler, Bio-Rad Laboratories, Hercules, CA) are described (**Table S2**). In the second round of amplifications, PCR products were diluted 1∶40 (v/v) in water. kDNA primers were substituted for the nested sets: S35 and S35 antisense, and the same L1 primers. In the third step, PCR products of *tp*TAIL-PCR 2 were diluted 1∶10 (v/v) in water and the LINE-1 primers were combined in the reaction with S67 antisense or S36. PCR products of the last amplification that hybridized with the kDNA probe were cloned directly into pGEM-T Easy Vector (Promega, Madison, WI). Clones selected by hybridization with the kDNA probe were sequenced commercially. Validation of the *tp*TAIL-PCR was determined in a mix of 200 ng DNA from two Europeans used as controls spiked with 300 pg of *T. cruzi* kDNA. The gold standard validation *tp*TAIL-PCR test was carried out with equal amounts of DNA from the ATCC U937 macrophage line. The temperatures and amplification cycles were the same used for the test patient DNA.

### Fluorescent *In Situ* Hybridization

Spreads of peripheral blood mononuclear cells from Chagas patients, their progeny, and control individuals were obtained after 3 h co-culture in the presence of 50 µg/ml of colchicine. Interphase and metaphase chromosomes were collected on glass slides and processed for *in situ* hybridization [20]. After rinsing three times with 2X SSC, slides were air-dried and incubated with RNase I (100 µg/µL) for 1 h at 37°C. Slides were rinsed twice in 2X SSC and dehydrated with 70%, 90% and 100% changes of cold ethanol at −20°C for 5 min each. Metaphase chromosomes were denatured in 50% formamide for 5 min at 92°C, and used immediately. The probes were labeled by a chemical reaction described in the Molecular Probes protocol (Invitrogen). Probes (4 ng/µL) were combined in hybridization buffer supplied with the dyes, denatured for 10 min at 72°C in 50% formamide in 2X SSC, and kept on ice for 30 min before application to the slides. The DNA probe (10 µL) was mixed with 70 µL of hybridization buffer, applied to the specimen slide, covered with a cover slip, and incubated in a humidified chamber at 37°C overnight. The slides were washed for 30 sec in 0.4X SSC/0.3% NP-40 at 73°C and allowed to incubate in this solution for 2 min, and again washed in 2X SSC/0.1 NP-40 at room temperature for 1 min. The slides were washed quickly in distilled water, and allowed to air dry in the dark. Two chromosome paints were obtained with the FISH Tag DNA Multicolor Kit (Molecular Probes, Invitrogen, CA). The Alexa Fluor 488-FITC was used to label the kDNA probe, and Alexa Fluor 532-CY3 was used to label either the LINE-1 clone 198 probe or the Tcz nDNA probe. The specimens were counterstained by incubation with Hoechst 33342 (Invitrogen, CA) for 5 min at room temperature; one drop of SlowFade antifade reagent (Molecular Probes) was dispensed on the glass slide, covered with cover slip, and imaged [71]. The spatial distribution of the fluorescent probes was observed using confocal laser-scanning microscopes (Leica SP5 or Zeiss 5-Live, Heidelberg, Germany) and images were collected simultaneously.

### Data Analyses

Sequence analyses were made by BLAST and alignments by CLUSTALW. The expected scores (e-values) were recorded for statistical significance (p<0.001). Localization of transposable elements in chimeric sequences was achieved by GIRI (http://girinst.org/censor/index.php). CLUSTER analyses were used to determine family structure of the kDNA minicircle sequences present in the human genome, and distances were obtained according to the Sørensen-Dice coefficient and grouping by the average linkage method, UPGMA.

## Results

### Epidemiological Features of the Population Studied

The study population consisted of 87 volunteers. Patients 1, 2, and 3 had parasitological demonstration of *T. cruzi* infection. The remaining 84 individuals belonged to five families (**a**, **b**, **c**, **d**, and **e**). In these patients cryptic *T. cruzi* infections were detected in the family heads and in some descendants by ELISA, indirect hemagglutination, and immunofluorescence examination, consistently showing specific anti-*T. cruzi* antibodies [7]. These results matched those obtained by standard PCR amplification of *T. cruzi* nDNA with the exceptions of cases 69 (family **d**) and 74 (family **e**) that showed nDNA signatures in the absence of specific antibodies. The family compositions included members born at distant locations in the major ecosystems throughout Brazil. The geographical origins of the families represent distinct ecosystems within Brazil **(** **Figure 1** **)**. Families **a**, **b**, and **c** were triatomine exposure-free for the previous three decades. Families **d** and **e** lived in contact with triatomine transmitters of *T. cruzi*. In addition, Chagas-free individuals (2 males and 3 females) born on the European Continent who had never been in contact with triatomines served as controls.

The epidemiological backgrounds of the families by immunological and nucleic acid tests defined three groups of patients: 1) live infection anti-*T. cruzi* antibodies and/or with nDNA and kDNA signatures; 2) kDNA signatures in the absence of anti-*T. cruzi* antibodies; 3) anti-*T. cruzi* antibody-free and parasite DNA-free. Having defined these profiles we asked whether the parasite DNA could be retained in the host genome.

### Targeting Primer TAIL-PCR (*tp*TAIL-PCR) Amplification of *Trypanosoma cruzi* kDNA Integrations in the Human Genome

**Figure 2:**
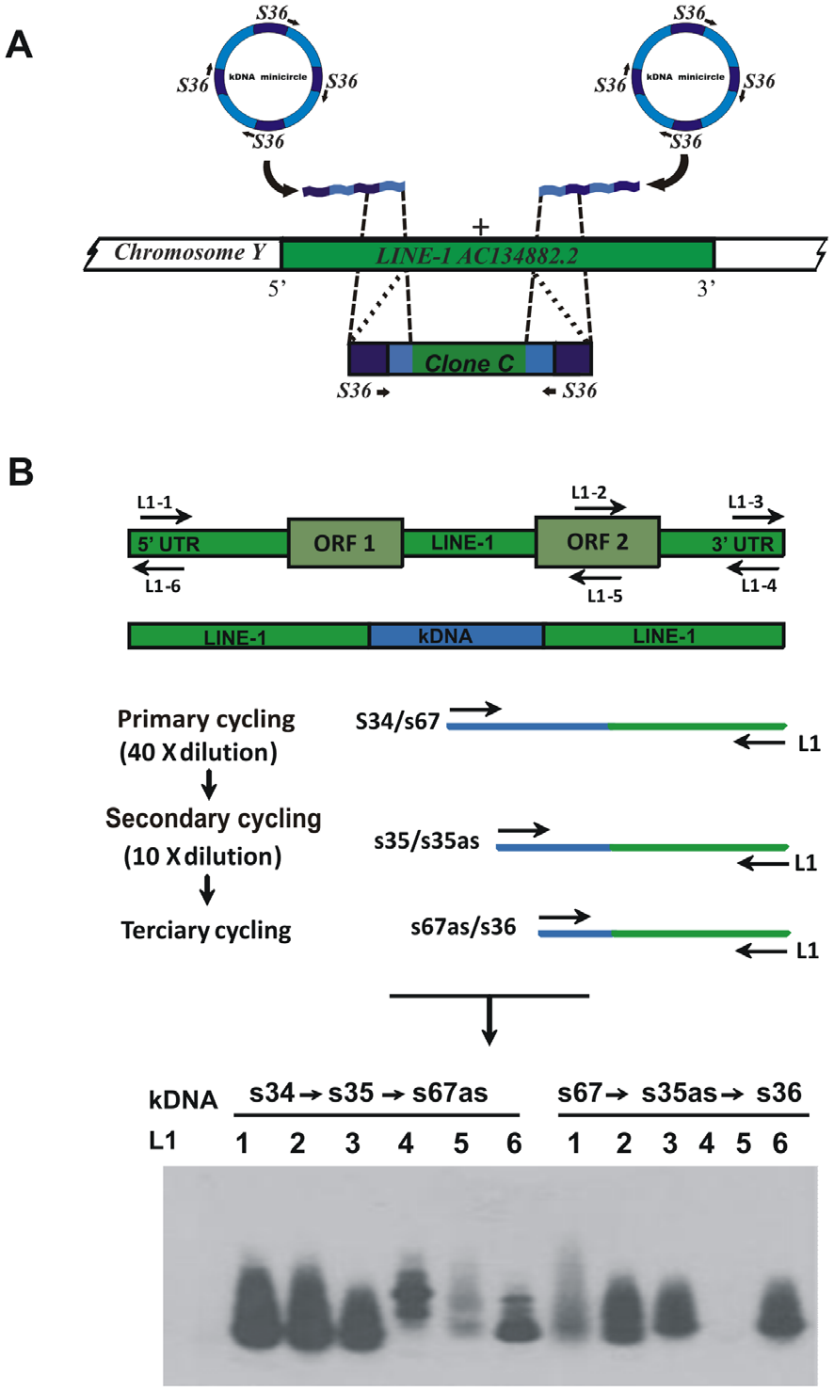
The modified *tp*TAIL-PCR used to detect *Trypanosoma cruzi* minicircle integrations into the human genome. (A) Truncated fragments of minicircles found in the LINE-1 copy at chromosome Y [20]; clone C showing conserved region annealed primer S36 (dark blue) at both ends followed by minicircle variable regions (light blue) and the LINE-1 sequence (green). (B) Targeted LINE-1 primers were used in combination with kDNA primer sets shown on top of the gel. The nested PCR reactions performed sequentially amplified target kDNA-host DNA sequences. The tertiary cycling products hybridizing with kDNA kCR probe on blots of 1% agarose gels were cloned and sequenced.

Previous documentation of LkDT was weak due to technical limitations. This problem was overcome by employing a *tp*TAIL-PCR strategy. Initially, the possibility that kDNA minicircles could integrate independently in proximity to LINE-1 elements was explored [20]. The amplification of host DNA was obtained in five instances (GenBank numbers AAF002199 to AF002203) using primer sets annealing to the flanking kDNA at both ends (**Figure 2A**). Annotations showing independent kDNA integrations in LINE-1 were analyzed, and representative sequences were aligned to obtain probes targeting the hotspots of DNA integration (**Figure S1**). Then, a modified *tp*TAIL-PCR was designed (**Figure 2B**) with the use of host-derived DNA probes (**Table S1**). Three cycles of amplification were used with temperature variation (**Table S2**). In each of these cycles the amplification products from the Chagas patient showed progressively increasing levels of hybridization with the cloned kDNA constant region probe (**Figure S2A**). The *tp*TAIL-PCR amplicons were cloned and sequenced, revealing kDNA-host DNA chimeric sequences (**Table S3**). The reliability of these findings stemmed from the sensitivity of the nDNA technique used, which is 10-fold higher than PCR using the kDNA-specific primers [69], [70]. In a control experiment, template DNA from infection-free volunteers showed no amplification after the third amplification cycle (**Figure S2B**).

The *tp*TAIL-PCR approach was successful in demonstrating minicircle integration into the genome of chronic Chagas patients (**Table S3**). Patients 1, 2, and 3 yielded independent LkDT events (emb|FM207254-to-FM207273) targeting retrotransposable elements at various chromosomes with significant e-value scores. These patients showed a minimum of four and maximum of eight independent kDNA integration events in various chromosomes. The combination of kDNA primer sets with LINE-1 5′-UTR-derived probes yielded 65% amplicon clones showing chimeric sequences. The e-values attained compared with minicircle and, separately, with the host flanking DNA ranged from 2.0e^−7^ to 2.0e^−58^, and from 2.0e^−14^ to zero, respectively. In addition, the combination of kDNA primer sets with LINE-1 ORF-2 region-derived probes yielded 100% amplicon clones showing kDNA-host DNA chimeras, for which almost complete homology (98% to 100%, e-values  =  zero) was observed. All these scores were significant statistically (p<0.001).

Some independent kDNA integration events linked stretches of chromosomes 14 with 10 (patient 2, FM207264), X with 10 (patient 3, FM207273), and 13 with 7 (patient 2, FM207263), suggesting mosaic recombination. In patient 3, kDNA integration in the 3′ end of the CLEC5A ORF (gb|AC073647.9), which linked to LINE-1 at the chromosome 7 q.33 locus, could produce a CLEC5A knockout (FM207269). This gene encodes a member of the C-type lectin/C-type lectin-like domain (CTL/CTLD) superfamily with diverse functions, such as cell adhesion, cell-cell signaling, glycoprotein turnover, and pro-immune response-mediated pro-inflammatory cytokines [72].

The validation protocols consisted of cloning and sequencing *tp*TAIL-PCR amplifications from a mix of control DNA with *T. cruzi* kDNA. In these replicate experiments the kDNA primer sets combined with L1-1 to L1-6 were used, and the amplification products obtained were hybridized with the kCR probe. Forty-one clones were sequenced; revealing kDNA stretches only (mean size 398±264 nts, **Table S4**). In similar validation experiments, ATCC macrophage DNA was mixed with kDNA, and the amplification products yielded exclusively kDNA sequence (**Figure S2C**). Furthermore, *tp*TAIL-PCR amplification products from ATCC macrophage DNA did not hybridize with the kCR probe (**Figure S2D**).

### Lateral kDNA Transfer (LkDT): *Trypanosoma cruzi* Minicircle Sequence Integration into the Human Somatic Cell Genome

**Figure 3:**
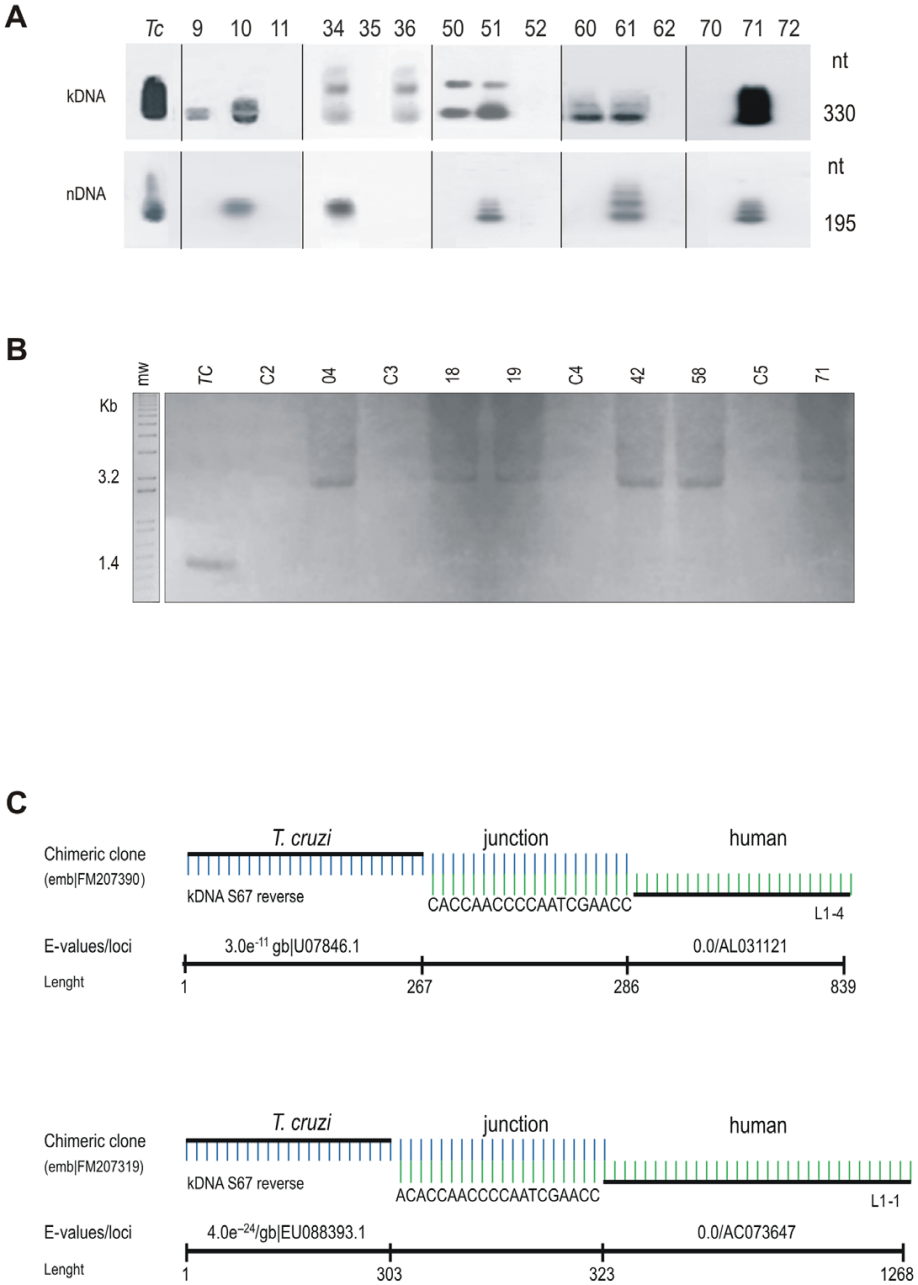
LkDT in somatic cells suggests mechanism of kDNA integration by homologous recombination. (A) Representative PCR amplifications of *T. cruzi* kDNA and nDNA. kDNA and nDNA signatures indicate active infections and LkDTs, while only kDNA indicates LkDT. Amplification products obtained with kDNA (S35/S36) and nDNA (Tcz1/Tcz2) primer sets hybridized with specific internal probes S67 and Tcz3, respectively, on blots of 1% agarose gels. The numbers above each lane indicates the study case. Tc, *T. cruzi*. Patients 10, 34, 51, 61 and 71, showing nDNA and kDNA signatures, harbor active *T. cruzi* infections. Patients 9, 36, 50 and 60, with only the kDNA signature, may carry inherited integration events. (B) Southern hybridization of integrated kDNA revealed by a minicircle (kCR) probe. A 0.8% agarose gel was used to analize *Eco*RI-digested DNA from Chagas patient blood mononuclear cells. Top numbers indicate family founders who had the kDNA integrations in LINE-1 on chromosome X (Table S3). DNA from uninfected human donors (C2 to C5) and from *T. cruzi* were included as controls. (C) Schematic representation of microhomology-mediated end-joining kDNA minicircle integration into retrotransposon LINE-1. Patients 71 (top) and 24 (bottom) show AC-rich intermediates involved in recombination. Each patient showing nDNA and/or kDNA footprints yielded at least one chimeric sequence.

The rate of kDNA integration into the peripheral blood mononuclear cells of the human population cohort was investigated using *tp*TAIL-PCR to amplify DNA templates from individuals belonging to the five families studied. *T. cruzi* DNA amplifications were obtained from family members for whom nDNA and kDNA signatures were detected (**Figure 3A**, and **Figure S3**), yielding sequences (accession numbers: emb|FM207274 to FM 207410) with minicircle footprints on several chromosomes (**Table S3**). Among the 466 clones, 296 (63.5%) chimeras were found with kDNA flanked by host DNA, while 138 (29.5%) showed only kDNA, and 32 (7%) bore only host DNA. A total of 154 unique integration events were detected in 55 individuals in families with accessible templates. The mean clone length was 617±267 nts with kDNA comprising 326±127 nts and host DNA 313±231 nts. The main targets for kDNA integration in the human genome were LINEs in 65% of the LkDT events. Also, integrations were present in coding regions containing short repetitive elements compatible with those found in retrotransposable elements. These LkDTs were distributed among various loci on different chromosomes. Multiple integrations were observed in many cases (mean 3±2), and a maximum of 10 independent events were disclosed on six different chromosomes in the male head of family **b** (**Figure 1**).

Phylogenetic analysis of the integrated minicircle constant and variable sequence stretches revealed high heterogeneity, as expected given the source material if the CSB is the point of recombination. A dendrogram (**Figure S4**) resolved several sequence classes, with no major branch emerging. The heterogenous nature of the tree indicates that no particular minicircle sequence class predominates in LkDT.

Minicircle sequences distributed throughout the five families showed increasing genetic diversity resulting from unaccountable events, and new genes and pseudogenes were observed. Blastx analysis showed 114 ORFs deduced from chimeric sequences (**Table S3**) observed with the potential for the translation of chimeric proteins, some of which showed significant similarities to *T. cruzi* proteins as well as to host cell proteins.

In summary, multiple LkDT events were detected in 25 patients (29.8%) with active *T. cruzi* infections. Furthermore, we detected VkDT in 29 F1 and F2 uninfected progeny (34.5%) from the five families that were free of nDNA and of specific anti-*T. cruzi* antibodies as assayed by ELISA (**Figure 1** **, and Figure S3**). Thirty individuals (35.7%) in the study population and five controls of European origin were both nDNA- and kDNA-free. Southern analysis of Chagas patient DNA with a kDNA probe revealed specific kDNA bands, while no control DNA from Europeans hybridized with the kDNA constant region probe (**Figure 3B**).

Chagas patient DNA served as templates for conventional PCR amplification of exogenous DNA integration. The amplifications that were obtained using the kDNA primers combined with the L1-1 to L1-6 primer sets contained kDNA stretches (33 of 41 clones, 80.5%), host DNA fragments (9.75%), and four clones (9.75%) revealed kDNA flanking host DNA (**Table S3**). These results were anticipated as each integrated linear kDNA stretch with constant 5′ and 3′ regions annealed with primer sets in opposing directions [20]. In control experiments, the DNA templates from *T. cruzi*-free individuals and from the ATCC macrophage line yielded no amplification. Depending on the combination of primer sets used, *tp*TAIL-PCR showed that from 65% to 100% of the clones had kDNA flanking host DNA, while conventional PCR showed kDNA flanking host DNA less than 10% of the time.

### Microhomology-Mediated End-Joining Mediates kDNA Minicircle Integration

A preference of LkDTs for repetitive sequence elements shared by minicircles and retrotransposable elements was observed. In each of the four conserved regions of *T. cruzi* minicircles, CSBs are associated with replication, transcription, and recombination [18], [19]. The insertion of kDNA at unique CSB-similar AC repeats shared by both minicircles and retrotransposable elements suggested integration hotspots (**Figure 3C**). The structural features of sequences carrying LkDTs showed intermediate stretches of six to twenty two nt sharing alignment similarity between minicircle and host DNA. In the LkDTs 942 AC-rich repetitions were counted that included kDNA minicircle (67%) and LINE (33%) sequences. Sequence alignments and absolute frequencies are shown in **Figure S5**. Five main CA repetitions (CCCAAAACCA/CCCAAAACC/ACACCAACCCCAA/ACCAACCCC/CCAACCCCAA) were present consistently at the junctions.

### Vertical kDNA Transfer (VkDT): *Trypanosoma cruzi* Minicircle Integration into the Human Germ Line

*T. cruzi* multiplies inside male or female germ-line cells [3]. The interactions between *T. cruzi* and growing stem cells from a 2.5-day-old zygote are illustrated in Video S1. Parasite invasion and multiplication in goniablasts is consistent with kDNA minicircle integration that could be transferred vertically to progeny [3]. The suspicion of VkDT stemmed directly from 29 progeny of Chagas patients where kDNA was detected in somatic cells that lack nDNA. Thus host sexual reproduction could streamline kDNA inheritance, leading to the investigation of this possible source of genetic diversity and speciation.

**Figure 4:**
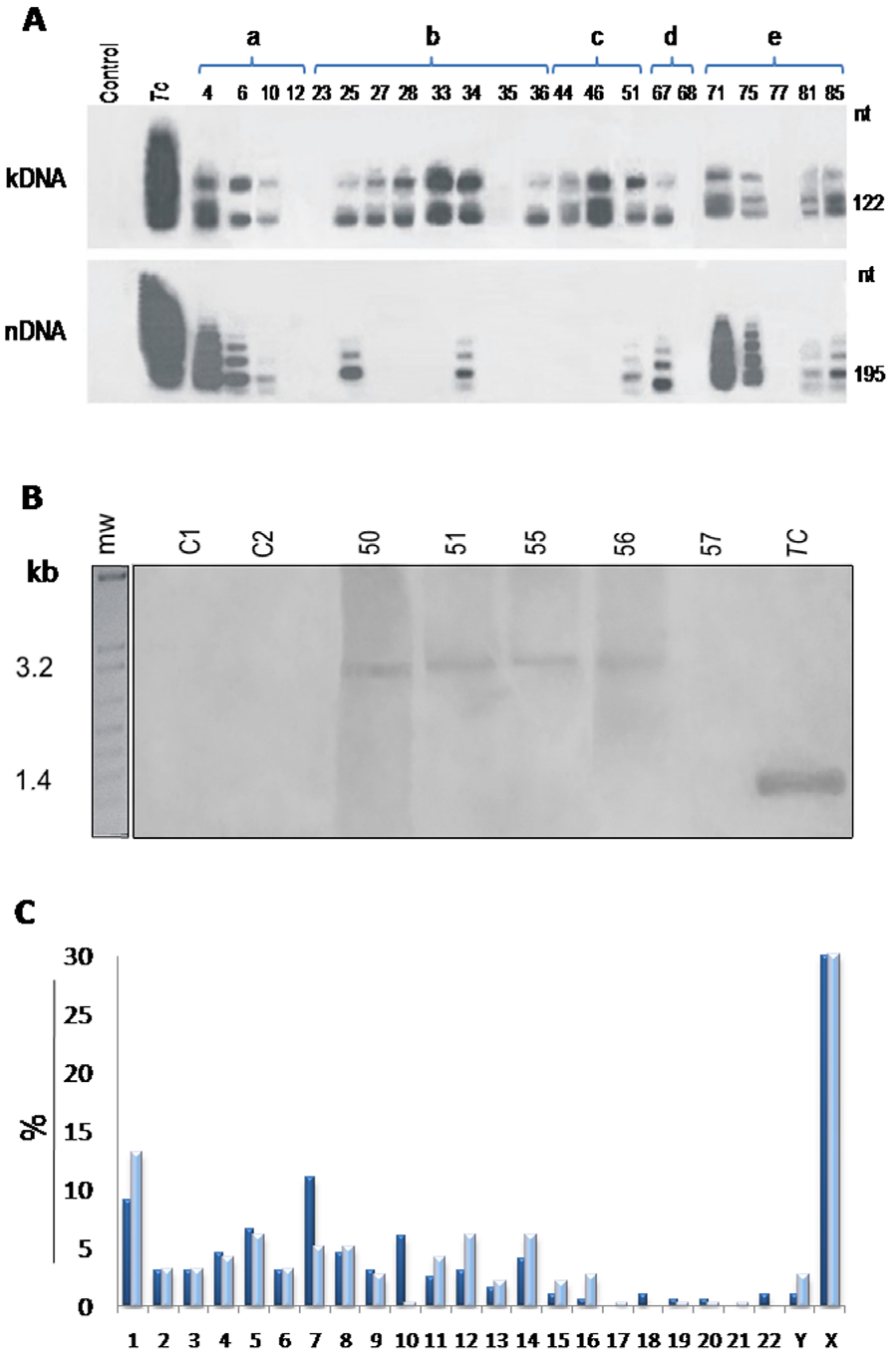
Tracking kDNA into germ line cells, and distribution of somatic and germline cells integrations into chromosomes. (A) Representative PCR amplifications of *T. cruzi* kDNA and nDNA from male gametes, obtained with kDNA S35/S36 and nDNA Tcz1/Tcz2 primers, and hybridization to a blot of a 1% agarose gels with the kCR probe. Top numbers indicate study cases. nDNA and kDNA signatures indicate active infections and LkDTs, while kDNA indicate VkDT. (B) Southern blot hybridizations of *Eco*RI digestions of human DNA with a kDNA-specific probe on blots of 0.8% agarose gels. Top numbers indicate family **c** F1 progeny (patient 50) married to Chagas case patient (51) showing kDNA integrations in LINE-1 on chromosome X. The DNA from the parents and from two progeny (patients 55 and 56) formed 3.2-kb bands, whereas DNA from progeny 57 and from uninfected patients (C1 and C2) did not. Tc, *T. cruzi*. (C) Frequency of kDNA integrations in chromosomes of somatic (dark blue) and of germ-line (light blue) cells.

Germ line cells from 20 F1 and F2 volunteer sperm donors provided the template material for amplification of nDNA and kDNA (**Figure 4A**). Both of these markers were positive in nine samples, kDNA alone was detected in six samples, and five were nDNA and kDNA-free. The positive nDNA signal indicated that *T. cruzi* cells were present in the sample, and thus could be sexually transmitted. *tp*TAIL-PCR-generated sequences were shown in Table S3. kDNA integrations were found at several loci: 79% of 149 clones showed kDNA flanked by host DNA, while 10% showed kDNA alone, and 11% bore only host DNA. The haploid cell-kDNA amplifications from 15 individuals yielded 106 novel mutation events (accession numbers: emb|FM207148 to FM207253). The main targets for kDNA integration in the male gametes were LINEs (72.5%); in 9% minicircle sequences targeted coding regions, while 4.5% were found in non-autonomous retrotransposons and 14% could not be identified. Multiple integrations were seen in many cases (mean 6.1±2.7), and a maximum of 11 independent events were captured in patient 4.

VkDTs were detected in the germ-line cells of nine males with active *T. cruzi* infections and in six infection-free F1 and F2 progeny within the population studied. An example of VkDT (**Figure S6A**) is shown explicitly by the alignment of chimeric sequences derived from somatic cells (FM207366) and from gametes (FM207220) of a father (patient 51) and from the somatic cells of his daughters (Patients 55 and 56, respectively, FM207368 and FM207370). The validation of these findings was provided by Southern analysis of *Eco*RI digestions from parents 50 and 51, and their daughters 55 and 56 with VkDT, but no nDNA nor specific anti-*T.cruzi* antibodies (**Figure 1**, and **Figure 4B**).

Multiple events of kDNA integration in the parents and descendants of the five families were documented. The distribution of LkDT and VkDT among the chromosomes is summarized (**Figure 4C**). The highest insertion frequency was found in chromosomes X, 1 and 7. The differences were proportional to the concentration of LINE-1 copy numbers on the X chromosome [73], where 18% of the LkDTs were observed, as well as 26% of the VkDTs, integrated into LINE-1 at nt 77363 of locus AL732374.14. The bioinformatics analyses showed that a 3′-host LINE consensus sequence in 33 clones annealed exclusively at locus AL732374.14, representing a type-specific hotspot in chromosome X (**Table S3**, and **Figure S6B**).

DNA templates from the kDNA-positive/*T. cruzi*-infection free progeny were subjected to conventional PCR amplification to query DNA integration. The amplifications obtained with assorted combinations of primer sets for specific annealing with kDNA and LINE-1 revealed kDNA stretches (82.4%) and host DNA fragments (5.9%). Furthermore, four clones (11.7%) showed kDNA integrated into host DNA: two integrations in chromosome 9 locus AL162420.131, and two in chromosome X locus AL732374.14 (**Table S3**). Consistent with previous results, no amplifications were obtained with DNA from control individuals or from ATCC macrophage line DNA.

### Co-localizing Minicircle Integration with LINE-1 in Whole Chromosomes

**Figure 5:**
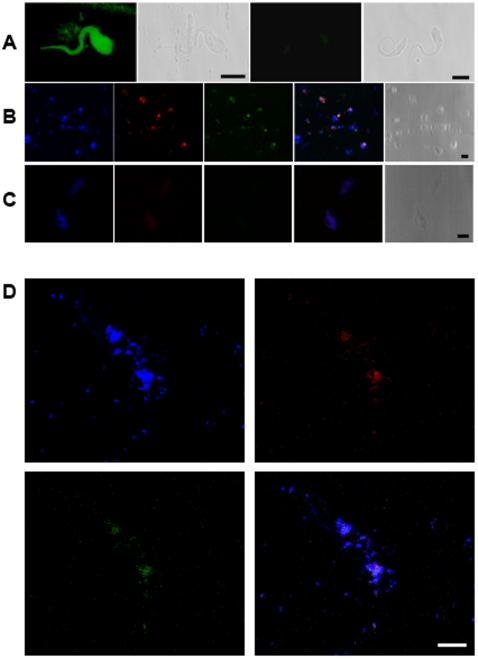
Co-localizing *Trypanosoma cruzi* kDNA sequence in metaphase plate chromosomes from human blood mononuclear cells. kDNA integrations were co-localized in metaphase plates of nine patients from families **a**, **b**, and **c** with the 350-bp probe resulting from *Nsi*I digest of wild-type kDNA, and with the LINE-1 clone 198 probe. (A) *T. cruzi* epimastigote fluorescein-labeled antibody (green) from Chagas patient serum (left) and control non-chagasic serum (right). The parasite silhouette is seen by digital image of contrast. B) *T. cruzi* DNA (Hoechst in blue), nDNA (Cy3 in red) and kDNA (FITC in green). The merged panel showed the parasite DNAs positioned with the digital image of contrast. C) *Leishmania (viannia) braziliensis* Hoechst stained DNA (blue); in absence of staining red (Cy3-LINE) and green (fluorescein-kDNA), respectively, with the 350-bp kDNA and with the LINE clone 180 probes. The merge was not positioned with the digital image of contrast. D) Metaphase chromosomes (blue), LINE-1 (red) and kDNA (green). The merge positioned with the digital image of contrast. Scale bars  = 5 µm.

In order to confirm integration of kDNA insertion in LINE-1 on various chromosomes, *in situ* hybridization was carried out on host DNA in the metaphase plate of blood mononuclear cells from Chagas patients and progeny, using the LINE-1 clone 198 probe (**Figure S1**) and the parasite probe derived from an *Nsi*I digestion of kDNA. LINE-1 and kDNA probes were co-localized on several chromosomes on metaphase plates. The LINE-1 clone 198 probe did not label parasite DNA in control hybridizations with *T. cruzi* cells. The kDNA probe co-localized with the LINE-1 hybridization using the specific probe on two chromosomes from Chagas patient 51 (**Figure 5**). The co-localization of kDNA and LINE-1 in chromosomes from Chagas patients and progeny was in agreement with previous *in situ* observations [20]. Specificity of the probes was supported by the absence of hybridization in *Leishmania braziliensis*, a distantly related kinetoplastid.

### Mosaic Recombination and Hitchhiking Minicircle Sequences

LkDT- and VkDT-induced-mutations were subject to mosaic-type recombination involving two or more DNA stretches from different loci (**Table S3**). Approximately 15% of the somatic cell mutations involved truncated stretches of minicircle interspersed with variable-length fragments hitchhiking from a second party locus. Over 38% of the germ-line mosaics showed variegated patterns linking three different loci with minicircle sequences. Ten percent of the germ-line mosaics had minicircle sequences and two host DNA stretches from different loci. Variable distances of interspaced microhomologies might explain these events of mosaic recombination or hitchhiking [20], [74].

### Genetic Drift and Social Heterosis

**Figure 6:**
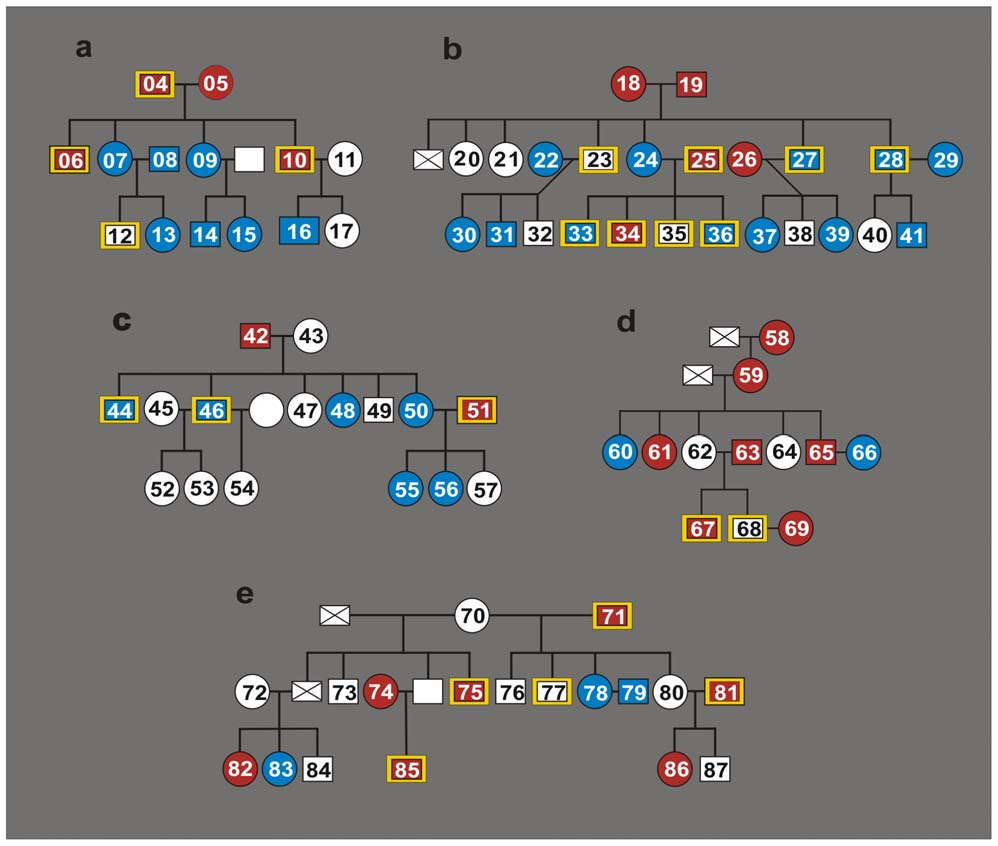
Phylogenetic patchwork resulting from LkDT and VkDT. Donors of diploid peripheral blood mononuclear cell DNA: Male, open square; female, open circle. Donors of haploid sperm cell DNA are indicated by yellow-edged square. Patients showing *T. cruzi* nDNA and kDNA are represented by red square or circle. Patients having integrated kDNA alone are identified by blue square or circle. Absence of a serial number means no biological sampling. A case deceased is indicated by crossed square. Volunteer samples were challenged by nDNA and kDNA amplifications withTcz1/Tcz2 and with S34/S67 primer sets, and blot hybridization with nDNA and kDNA probes. Each family member having nDNA and/or kDNA footprints yielded at least one chimeric sequence.

These results suggest that LkDT and VkDT events in the various age groups of the study population were subject to the forces of adaptive selection and genetic drift. The phenomenon of social heterosis may foster these processes by the mating of people from distant ecosystems creating increased genetic diversity. To visualize this effect, the integration data (**Table S3**) was used to generate a phylogenetic patchwork (**Figure 6**). kDNA mutations were present in 27 out of 41 (61.4%) of the F1 progeny and in 20 out of 33 (60%) of the F2 progeny.

The epidemiological history of the population studied (**Figure 1**) helps in the interpretation of the patchwork resulting from LkDT and VkDT. For example, the family **b** founders born in an Amazonian rain-forest ecosystem had F1 progeny 24, who married out-of-bloodline Chagas 25 patient from the coastal Atlantic forest. Parents 24 and 25 along with their F2 progeny 33 and 36 lived in a triatomine-free region where the possibility of insect vector-transmitted *T. cruzi* infection is negligible. Thus, a mix of *T. cruzi* populations might have contributed minicircle sequences to progeny 33 and 36. Furthermore, F2 progeny 34 bore nDNA and kDNA markers suggestive of a living infection. The phylogenetic patchwork of this family reveals that, in addition to out-breeding stemming from genetically diverse mate selection, LkDT and VkDT contributed to the increase in social heterosis, and should be included as yet another source of diversity [75].

### Minicircle Disruption of Host Genes

A gamut of LkDT events (12%) promoted rupture of 13 different ORFs (**Table S5**). In the genes PARP-1, Theta-14-3-3, CNTNAP2, and human lymphocyte antigen (HLA), respectively encoding ADP-ribosyltransferase, phosphoserine binding and signal transduction, adhesion molecules and receptors in vertebrate nervous system, and major histocompatibility complex class 1, sequence analyses showed significant homology with LINE-1 as well as another truncated element. An integration event at the RP11-156A1 locus of chromosome 2 revealed that kDNA appeared to rupture the ADAM 23 ORF involved in important cell functions related to development, fertilization, myogenesis and neurogenesis. LkDTs involving olfactory genes were detected in seven cases. Intriguingly, HLA-linked olfactory receptor (OR) genes appear to participate in olfaction-guided mate choice and potentiating diversity [76]. Possible functional alterations resulting from the rupture of genes is a tempting subject of investigation, focusing on population fitness and diversity. Furthermore, 10% of the LkDT events were detected in the non-autonomous retrotransposons called SINEs (Alu and MIR, 61%), MER, ERV, and MalR (39%), which repeatedly were found downstream of LINE-1. In 13% of the sequences matching human DNA templates, integrations occurred in various chromosomes at undetermined sites. In the presence of AC repetition-shared microhomologies present in truncated ORFs, these mutations may have hitchhiked from primary LkDT-LINE-1-promoted retrotranspositions.

## Discussion

In this report we show that *T. cruzi* minicircle sequences integrated into the genome of Chagas patients in five families from different ecosystems of Brazil. The specific integration sequences were obtained using a modified *tp*TAIL-PCR, employing targeting primers annealing to conserved regions of LINE-1, which demonstrated that kDNA sequences integrate consistently into retrotransposable elements within the human host genome. Also, Chagas patient progeny showing kDNA signatures had minicircle sequences integrated in their genomes in the absence of both nDNA footprints and specific anti-*T. cruzi* antibodies. The resulting host mutations showed various patterns of recombination. Somatic and germ-line genomic DNAs with minicircle integration events were confirmed by Southern analysis. Furthermore, LkDT- and VkDT-mutations were used to visualize phylogenetic patchworks depicting polymorphism and social heterosis. The transfer of minicircle sequences from *T. cruzi* to humans, an occurrance provoking genome growth and mosaic recombination, had the unexpected consequence of increasing genetic diversity, while generating resident gene knockouts by the primary integration or subsequent hitchhiking.

Minicircle integration in male gametes was documented in all five families. Likewise, female gametes played a role in the vertical transfer of kDNA. As an example, the F1 and F2 progeny in family **b**, patients 22, and 30 and 31, respectively, illustrate one of several instances of germ-line transmission through multiple generations (**Figure 6**). The pathological documentation of dividing *T. cruzi* in the theca cells of the ovary and in the goniablasts of the seminiferous tubes of the testes in acute Chagas disease [3] support these conclusions. This work complements and extends hypotheses presented in our previous studies [3], [9], [10], [20], [53].

**Figure 7:**
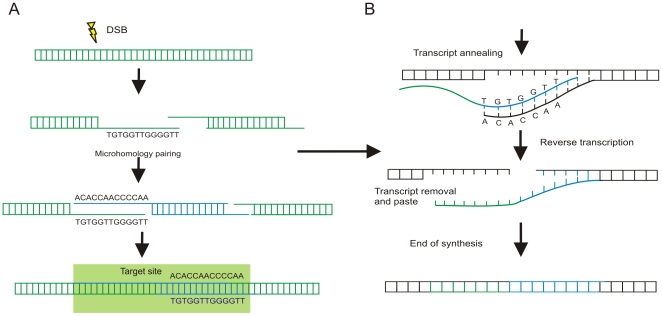
Model of *Trypanosoma cruzi* minicircle integration and replication in the human genome. (A) Infection-induced host DNA (green) double strand-break (DSB) and integration of kDNA minicircle sequence (blue) mediated by microhomology end-joining. (B) Replication of the kDNA-LINE sequence by target-primed reverse-transcription using the autonomous recombination machinery of the LINE-1. The kDNA sequence repeat received a specific cut, and free-end pairing with a first strand transcript (green). A complementary second strand transcript is made, which can transpose to different chromosomes.

Cryptic *T. cruzi* infections in members of five families were validated by the presence of parasite kDNA and nDNA footprints. F1 and F2 progeny showing only kDNA footprints had minicircle sequences integrated mainly into LINE-1 hotspots, but also in SINEs and in other retro-elements of the human genome. Among the important interactions of the intracellular pathogen and its host cell [50], decatenation of the minicircles prior to cell division may be crucial, because the four AC-rich regions, the CSBs, are exposed, matching the short complementary sequences in the host DNA [18], [19], [20]. These short AC-rich sequence stretches are widespread in kDNA-LINE chimeras identified in this study. The frequency of these repetitions links the kDNA sequences with the host DNA by a microhomology-mediated end-joining mechanism (**Figure 7**). This important topological feature of the minicircles could facilitate the mosaic recombination and hitchhiking observed in the genomes of Chagas patients and their progeny.

The abundance of kDNA insertions detected in blood mononuclear cells or ejaculate might be explained by the hundreds of thousands of copies of LINEs, SINEs, MER, ERV, and MalR retro-elements in the human genome. Thousands of minicircle copies may flood the host cell nucleus during an active *T. cruzi* infection. Hundreds of AC-rich junctions identified between minicircle and LINE sequences were encountered. The small AC-rich regions punctuated throughout non-autonomous retro-elements downstream of LINEs could be factors in promoting genomic instability [36], [40], [41], [44]. The occurrence of minicircles linked to LINE sequences via AC-rich microhomology-mediated recombination may augment endogenous levels of recombination, thus explaining the active generation of mosaic sequences and minicircle hitchhiking along with their associated retroposon, as seen in germ-line cells of Chagas patients and their progeny. Shuffling of the integrated minicircle-LINEs may open renewable sites for the accumulation of additional integration events. Likewise, proliferation of the kDNA-LINE chimeras via the endogenous retrotransposon machinery of precursor cells in the bone marrow and gonads may explain the abundance of LkDT in the families studied. The occurrence of LkDT in the course of other chronic infections by different intracellular eukaryotic pathogens remains to be documented. This hypothetical generality may be as commonplace as in viruses, the ultimate minimalist parasites.

The suggestions that *T. cruzi* infection contributes to accumulation of LkDTs, and that genotypic and phenotypic alterations would drive subsequent autoimmune destruction of “self” target tissues [3], [20] provided the stimuli for our efforts. A scenario that connects the LkDT-derived chimeric proteins, which are not necessarily self-proteins, to the induction of immune intolerance awaits experimental description. Evidence for autoimmunity stemming from somatic genetic alterations that render newly differentiated effector cells insusceptible to elimination or inhibition by the host censorship mechanism is sought. Particular disease manifestations in the founders and in some of their descendants might be explained by genotypic alterations. Association of clinical manifestations of Chagas disease in patients with accumulation of the kDNA mutations and gene knockouts awaits epidemiological correlation. Complete sequencing of the genomes of Chagas patients with variable manifestations, such as cardiac or digestive system disease, to unravel fully the extent of kDNA-induced genotype modification may be a realistic experimental approach in the near future.

The concept of kDNA integration-induced genetic change of somatic host cells holds promise for the understanding of Chagas disease pathogenesis. A practical demonstration of the role that *T. cruzi* kDNA-induced mutation and genotypic-phenotypic alterations play in the rejection of target cells in Chagas disease requires a ‘clean’ parasite-free animal model. The use of outbred chickens hatched from *T. cruzi-*infected fertile eggs that become refractory to the infection before hatching partially satisfies this experimental requirement. Parasite-free chickens showing kDNA mutations develop cardiomegaly, whereby target heart cells are destroyed by effector immunocytes [3]. Expanding knowledge of the role played by autoimmunity in Chagas disease can determine the pathogenesis associated with rejection of heart cells in the progeny of congenic birds that bear kDNA mutations. It is hoped that these ongoing experiments will clarify familial genetic predisposition to the clinical manifestation of autoimmune disease.

Evolution by *trans*-species polymorphism leads to continual expansion within the biological system. The demonstration of active transfer of a mitochondrial DNA from *T. cruzi* to human hosts establishes the experimental grounds for this reasoning, and provides the basis for the intricate patchwork arising. In view of the growing concern about reduction of allelic polymorphism, possibly by natural selection [23], [24], [77] opposing the continuing expansion of genetic diversity, the inheritance of mutation loads to F1 and F2 progeny calls for a major conceptual change. The genetic drift of integrated kDNA mutations are consistent with the hypothesis that recombination, hitchhiking and social heterosis are active forces of evolution and speciation. The epidemiological history of our five families shows that minicircle sequences are not only inherited, but continue to spread through the progeny genomes by hitchhiking along with retrotransposition events mediated by LINE-1. In the context of the family founders having *T. cruzi* infections, the kDNA mutations present in 38.1% F1 and F2 progeny clearly are subject to genetic drift and selection. Thus, fixation of kDNA mutations in the human host genome and accumulating over time may influence the genetic structure of the population. While parasite populations show growing plasticity within their mammalian hosts [19], the intermingling of human populations increases host genetic diversity, and the parasite-host duet continues.

The transfer of mitochondrial DNA from *T. cruzi* to the human population will be significant broadly in population genetics. These infections have already touched millions of people in Latin America, approximately a million of whom are migrating continuously to other continents. Enzootic *T. cruzi* infections affect mammalian species in the Americas [3], and the prevalence of infection among wild mammals remains high [78]. Approximately one third of chronically infected individuals will die of Chagas disease, but usually when they are over 40 years of age; thus the disease does not interrupt the host reproductive process. Accumulation of kDNA mutations, recombination, and hitchhiking, with the associated genotypic and phenotypic modifications, likely will drive pathology. In this respect, Chagas disease manifestations may be an unintentional by-product of the parasite's intracellular life cycle and unusual mitochondrial DNA topology. The sheer mass of the minicircle DNA in the trypanosome genome may account for the transfer into the host, and the human population may be a patchwork of all the organisms to which it has ever been exposed.

### Conclusions

A modification of the TAIL-PCR, which consisted of using targeted primers directed against regions of the LINE-1 integration hotspot [20], allowed the capture of parasitic DNA integration into the human genome. The cloned kDNA-LINE-1 flanking regions yielded integration junctions that could be associated with both the parasite and the host. The LkDT or VkDT events were largely independent, as parasitic kDNA integrations could occur via germline or congenital transmission. The LkDT- and VkDT-induced genotypic and phenotypic alterations might explain the variability of some clinical manifestations of Chagas disease. Further studies are required to clarify the genetic basis of autoimmune lesions. Considering the consequences of parasite DNA acquisition in an evolutionary sense, vertical inheritance of integrated DNA and its subsequent drift may contribute to ongoing genetic diversity and speciation in the human population.

## Supporting Information

Figure S1 Host DNA probes targeting LINE-1 regions. CLUSTALW alignment of sequences from LINE-1 species, and definition of targeting probes annealing to 5′-UTR, ORF2, and 3′-UTR regions. Asterisks indicate LINE-1 present in ATM gene U82828.1, DMD gene U60822.1, and in BAC clones RP11-292P9 and RP11-44G14. L1-1 to L1-6 sequences orientation (arrows) is given.
(9.94 MB TIF)

Figure S2 tpTAIL-PCR control and validation experiments. (A) Template DNA from a Chagas patient. The tpTAIL-PCR yielded thermal asymmetric amplicons with increasing specificity after hybridization with radioactive kDNA probe on blots of 1% agarose gel. (B) Template DNA from control, uninfected donor. The tpTAIL-PCR amplification products did not hybridize with the specific kDNA constant region kCR probe. (C) tpTAIL-PCR validation experiment with a mix of kDNA and control macrophage DNA. The unique specificity of the amplification products was shown by hybridization with the radioactive kDNA constant region kCR probe. (D) Template DNA from control ATCC macrophage line DNA. The tpTAIL-PCR amplification products did not hybridize with the specific kDNA constant region kCR probe.
(9.98 MB TIF)

Figure S3 Signatures of *Trypanosoma cruzi* DNA in somatic cells of members from five families whose founders had active protozoan infections. PCR amplifications of *T. cruzi* nDNA and kDNA were obtained with kDNA (s35/36) and nDNA (Tcz1/2) primer sets, and hybridizations with the kCR probe. p, Pilot study with negative and positive controls. a-to-e, family members showing specific anti-*T. cruzi* antibody (see Figure 1) had kDNA and nDNA footprints, and harbored living infections. Family members showing only kDNA had it integrated in their DNA in the absence of infection.
(9.92 MB TIF)

Figure S4 Dendrogram showing genetic diversity of integrated kDNA minicircles. Black line, patients 1 to 3; blue, red, orange, green, and purple lines represent, respectively, families a to e. Each case showing nDNA and/or kDNA yielded at least one chimeric sequence.
(4.16 MB TIF)

Figure S5 Microhomologies present in host DNA and *Trypanosoma cruzi* minicircles. Multiple alignments of the short repeats present in LkDT events described in Table S3. The number in the right column shows how many times that specific repeat profile was found in the 154 chimeric sequences.
(9.95 MB TIF)

Figure S6 Lateral and vertical transfer of *Trypanosoma cruzi* kDNA sequences into human chromosome X locus AL732374.14. (A) LkDTs and VkDTs within a family. Alignments of minicircle sequences found in the genome of a father (emb|FM207366), and his daughters (emb|FM207368, and emb|FM207370), respectively, cases 51, 55, and 56, depicted in the phylogenetic patchwork, Figure 6. (B) LkDTs and VkDTs in LINE-1 at locus AL732374.14. kDNA integration events in this locus beginning at nucleotide 73363 of the clone RP13-444k19 (emb|AL732374.14) generated alignments of consensus sequences.
(9.82 MB TIF)

Table S1 Probes used in the tpTAIL-PCR amplifications.
(0.00 MB PDF)

Table S2 Thermal conditions for ptTAIL-PCR*.
(0.01 MB PDF)

Table S3 Transfer of minicircle sequences of kDNA from *Trypanosoma cruzi* to the human genome.
(0.14 MB PDF)

Table S4 Validation of the tpTAIL-PCR using different experimental protocols.
(0.03 MB PDF)

Table S5 Lateral transfer of *Trypanosoma cruzi* kDNA minicircle sequences provoking gene knock-out into the human genome.
(0.01 MB PDF)

Video S1 Interplays between *Trypanosoma cruzi* and a 2.5 days-old embryonic cells in vitro.
(10.17 MB AVI)
