# Supplementary figures and images for: Inheritance of DNA Transferred from American Trypanosomes to Human Hosts
Source: PLoS One. 2010 Feb 12;5(2):e9181. doi: 10.1371/journal.pone.0009181 (PMC2820539; doi:10.1371/journal.pone.0009181)

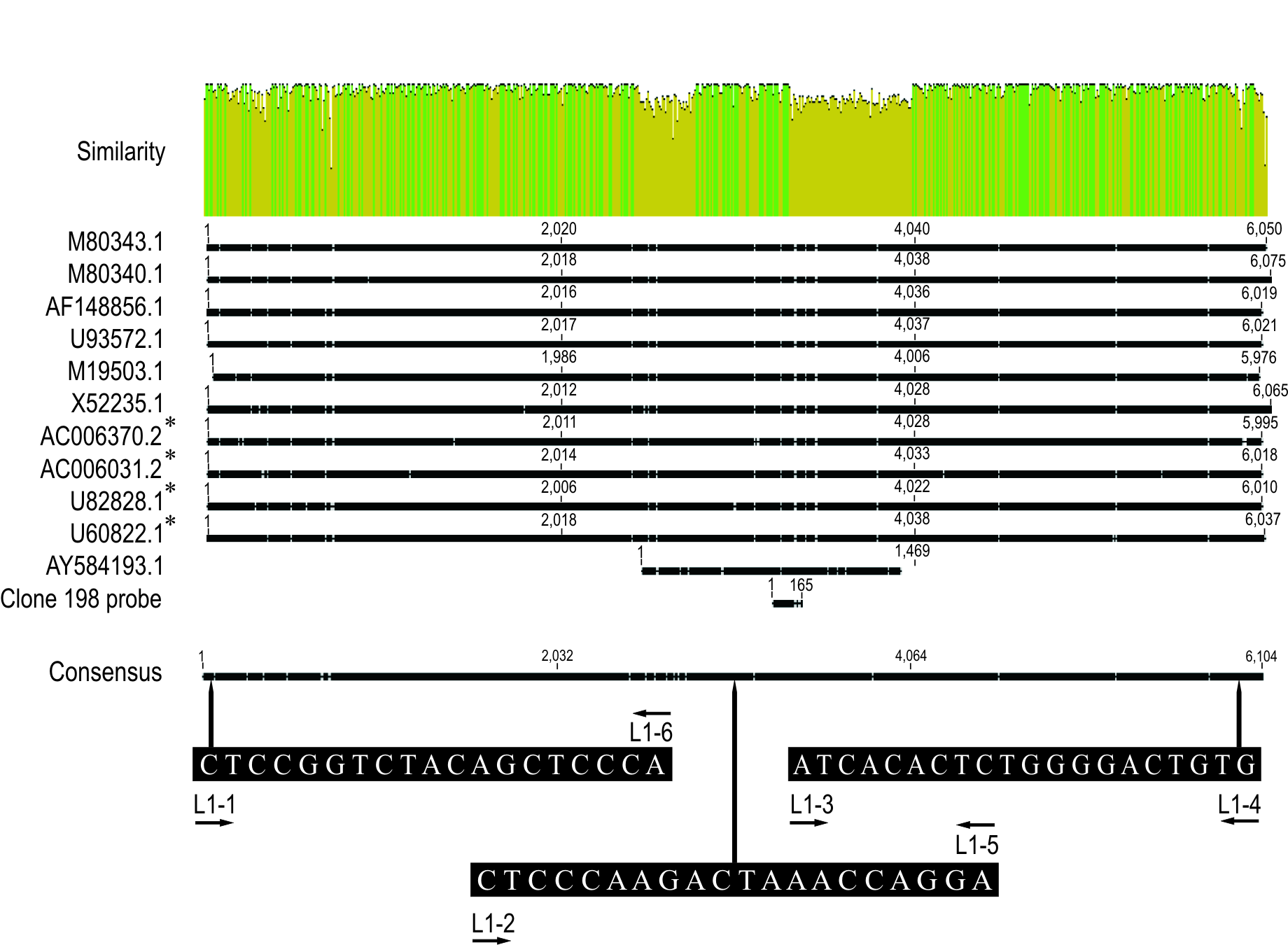

Supplement: Figure S1 — Host DNA probes targeting LINE-1 regions. CLUSTALW alignment of sequences from LINE-1 species, and definition of targeting probes annealing to 5′-UTR, ORF2, and 3′-UTR regions. Asterisks indicate LINE-1 present in ATM gene U82828.1, DMD gene U60822.1, and in BAC clones RP11-292P9 and RP11-44G14. L1-1 to L1-6 sequences orientation (arrows) is given. (9.94 MB TIF) [file pone.0009181.s001.tif]

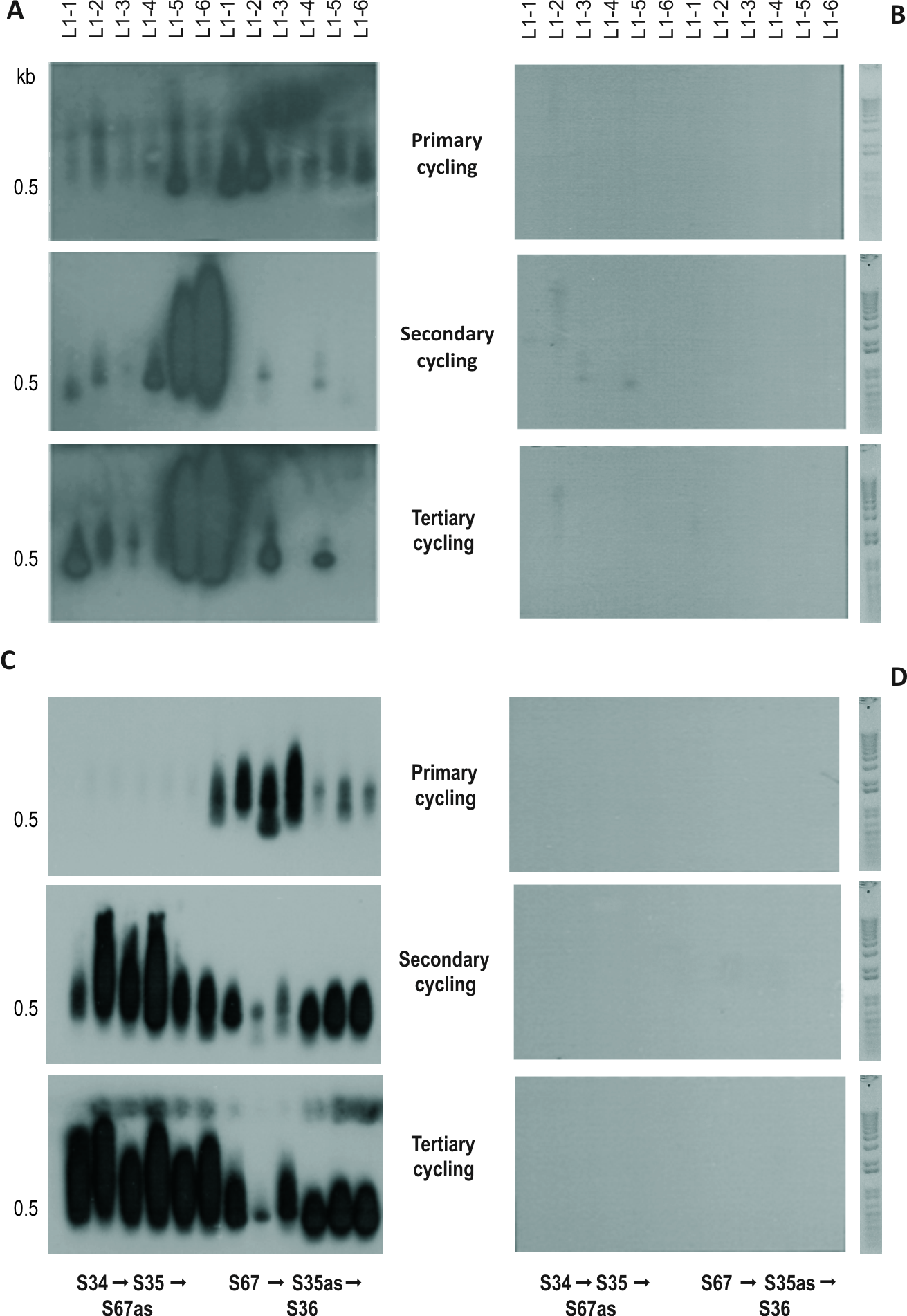

Supplement: Figure S2 — tpTAIL-PCR control and validation experiments. (A) Template DNA from a Chagas patient. The tpTAIL-PCR yielded thermal asymmetric amplicons with increasing specificity after hybridization with radioactive kDNA probe on blots of 1% agarose gel. (B) Template DNA from control, uninfected donor. The tpTAIL-PCR amplification products did not hybridize with the specific kDNA constant region kCR probe. (C) tpTAIL-PCR validation experiment with a mix of kDNA and control macrophage DNA. The unique specificity of the amplification products was shown by hybridization with the radioactive kDNA constant region kCR probe. (D) Template DNA from control ATCC macrophage line DNA. The tpTAIL-PCR amplification products did not hybridize with the specific kDNA constant region kCR probe. (9.98 MB TIF) [file pone.0009181.s002.tif]

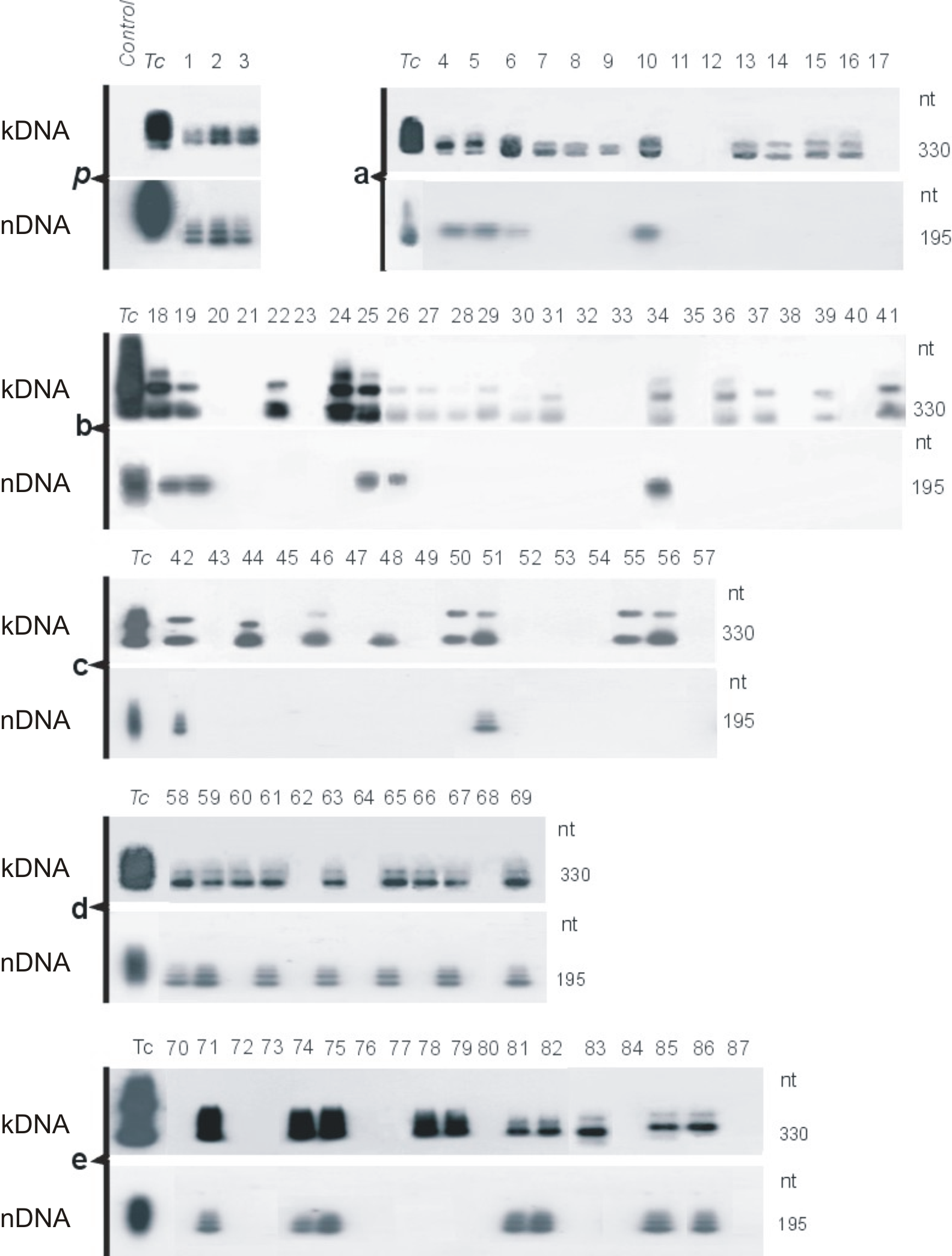

Supplement: Figure S3 — Signatures of Trypanosoma cruzi DNA in somatic cells of members from five families whose founders had active protozoan infections. PCR amplifications of T. cruzi nDNA and kDNA were obtained with kDNA (s35/36) and nDNA (Tcz1/2) primer sets, and hybridizations with the kCR probe. p, Pilot study with negative and positive controls. a-to-e, family members showing specific anti-T. cruzi antibody (see Figure 1) had kDNA and nDNA footprints, and harbored living infections. Family members showing only kDNA had it integrated in their DNA in the absence of infection. (9.92 MB TIF) [file pone.0009181.s003.tif]

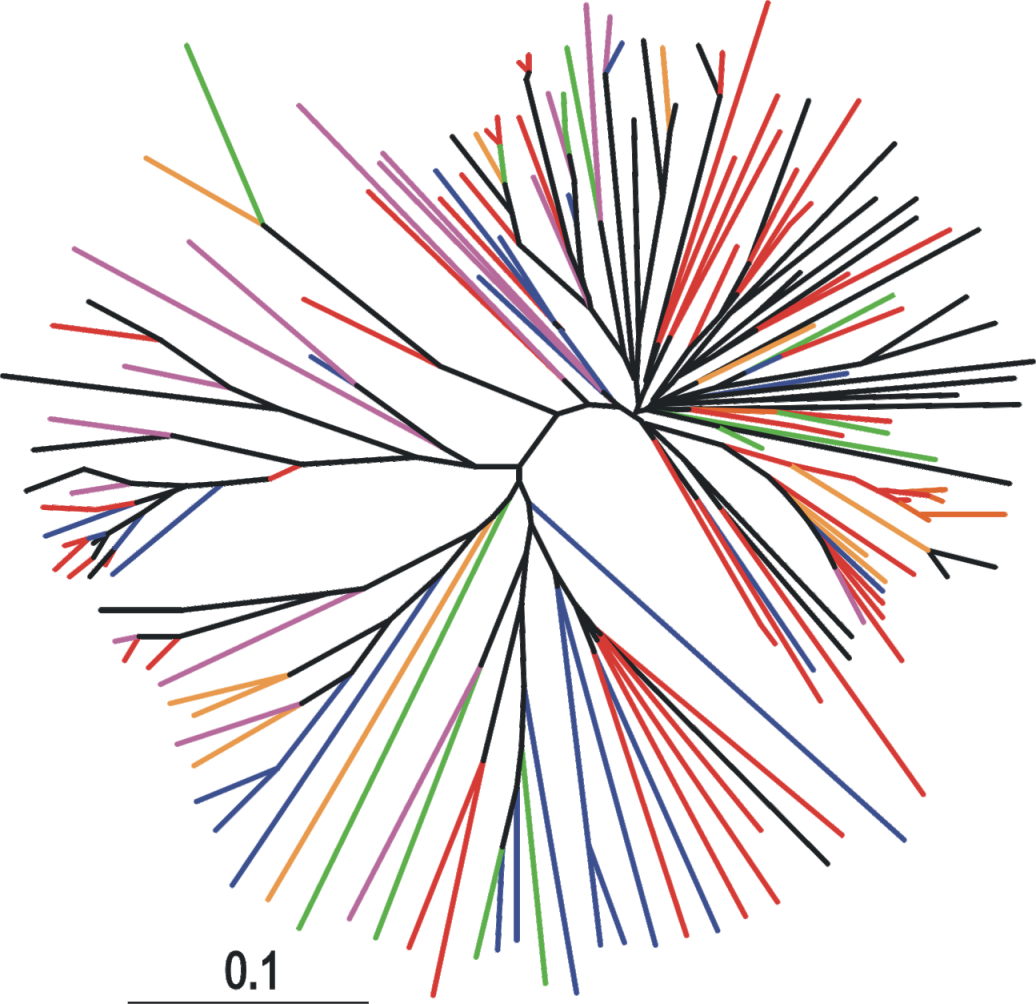

Supplement: Figure S4 — Dendrogram showing genetic diversity of integrated kDNA minicircles. Black line, patients 1 to 3; blue, red, orange, green, and purple lines represent, respectively, families a to e. Each case showing nDNA and/or kDNA yielded at least one chimeric sequence. (4.16 MB TIF) [file pone.0009181.s004.tif]

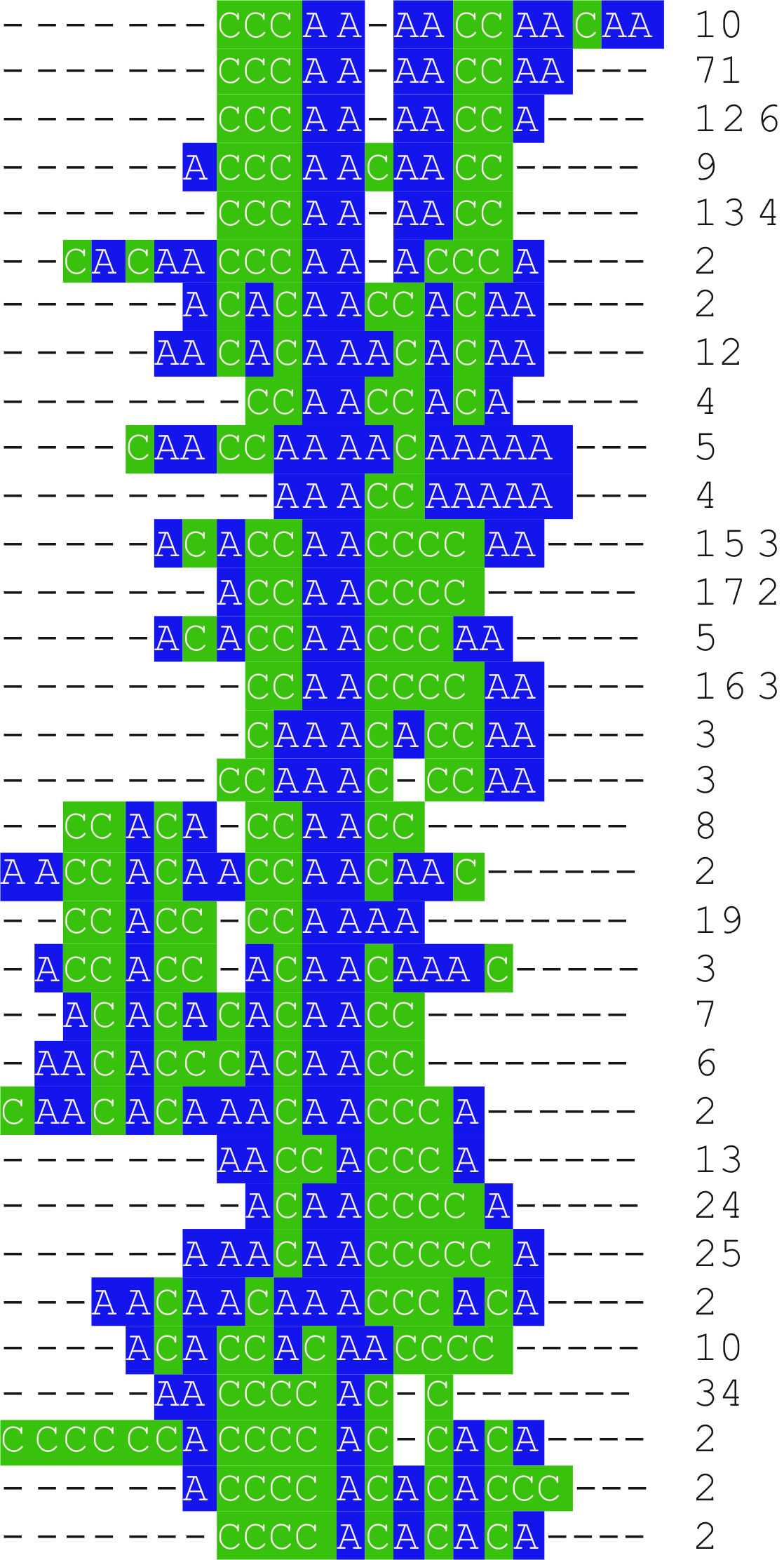

Supplement: Figure S5 — Microhomologies present in host DNA and Trypanosoma cruzi minicircles. Multiple alignments of the short repeats present in LkDT events described in Table S3. The number in the right column shows how many times that specific repeat profile was found in the 154 chimeric sequences. (9.95 MB TIF) [file pone.0009181.s005.tif]

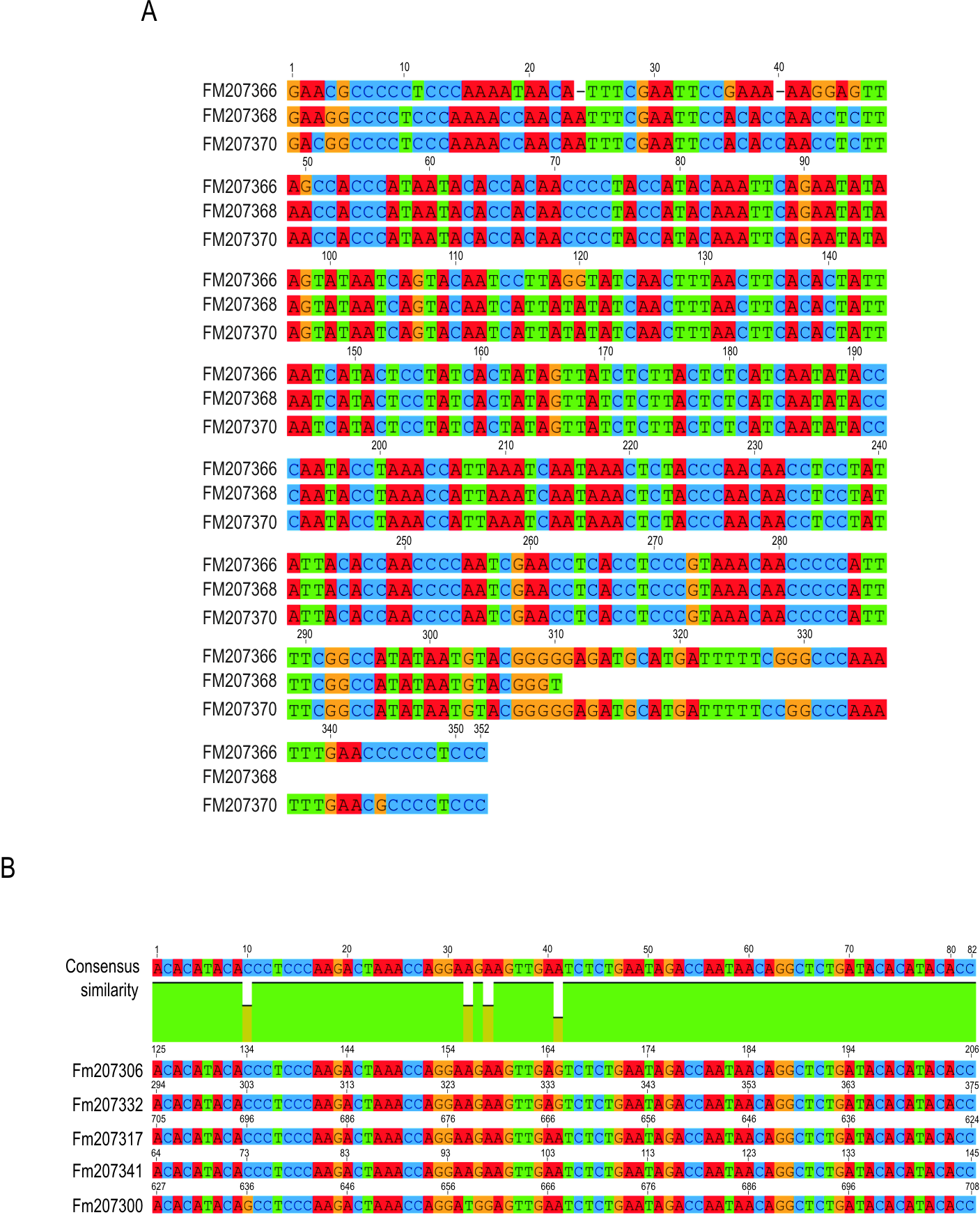

Supplement: Figure S6 — Lateral and vertical transfer of Trypanosoma cruzi kDNA sequences into human chromosome X locus AL732374.14. (A) LkDTs and VkDTs within a family. Alignments of minicircle sequences found in the genome of a father (emb|FM207366), and his daughters (emb|FM207368, and emb|FM207370), respectively, cases 51, 55, and 56, depicted in the phylogenetic patchwork, Figure 6. (B) LkDTs and VkDTs in LINE-1 at locus AL732374.14. kDNA integration events in this locus beginning at nucleotide 73363 of the clone RP13-444k19 (emb|AL732374.14) generated alignments of consensus sequences. (9.82 MB TIF) [file pone.0009181.s006.tif]
