# Supplementary material for: Inheritance of DNA Transferred from American Trypanosomes to Human Hosts
Source: PLoS One. 2010 Feb 12;5(2):e9181. doi: 10.1371/journal.pone.0009181 (PMC2820539; doi:10.1371/journal.pone.0009181)
Supplement: Table S1 — Probes used in the tpTAIL-PCR amplifications. (0.00 MB PDF) [file pone.0009181.s007.pdf]

Table S1. Probes Used in the *tp*TAIL-PCR Amplifications

**Primer**	**Target DNA**	**Sequence**	**Tm***
S 34	kDNA	5’ACA CCA ACC CCA ATC GAA CC 3’	57,9
S 67	kDNA	5’GGT TTT GGG AGG GG(G/C) (G/C)(T/G)T C 3’	60,1
S 35	kDNA	5’ATA ATG TAC GGG (T/G)GA GAT GC 3’	59,4
S 36	kDNA	5’GGT TCG ATT GGG GTT GGT G 3’	57,9
L1-1	LINE	5’CTC CGG TCT ACA GTC CCC A3’	65,6
L1-2	LINE	5’TCC CAA GAC TAA ACC AGG A3’	62,9
L1-3	LINE	5’ ATC ACA CTC TGG GGA CTG TG 3’	64,7
L1-4	LINE	5’ CAC AGT CCC CAG AGT GTG AT 3’	59,9
L1-5	LINE	5’ TCC TGG TTT AGT CTT GGG AG 3’	60,1
L1-6	LINE	5’ TGG GAG CTG TAC ACC GGA G 3’	63,0

* Tm = average annealing temperature ºC.
