# Supplementary material for: Inheritance of DNA Transferred from American Trypanosomes to Human Hosts
Source: PLoS One. 2010 Feb 12;5(2):e9181. doi: 10.1371/journal.pone.0009181 (PMC2820539; doi:10.1371/journal.pone.0009181)
Supplement: Table S2 — Thermal conditions for ptTAIL-PCR*. (0.01 MB PDF) [file pone.0009181.s008.pdf]

Table S2. Thermal conditions for *pt*TAIL-PCR*

Primary Reaction			Secondary Reaction			Terciary Reaction		
Step	Temp °C	Time (min)	Step	Temp °C	Time (min)	Step	Temp °C	Time (min)
**1**	94	4:00	1	94	4:00	1	94	3:00
**2**	94	0:30	2	94	0:30	2	94	0:30
**3**	64	1:00	3	64	1:00	3	45	1:00
**4**	72	2:00	4	72	2:00	4	72	2:00
**5**	Go to step 2	5X	5	94	0:30	5	Go to step 2	20X
**6**	94	0:30	6	64	1:00	6	72	7:00
**7**	25	2:00	7	72	2:00	7	4	∞
**8**	72	2:00	8	94	0:30			
**9**	94	0:30	9	45	1:00			
**10**	64	1:00	10	72	2:00			
**11**	72	2:00	11	Go to step 2	12X			
**12**	94	0:30	12	72	7:00			
**13**	64	1:00	13	4	4:00			
**14**	72	2:00						
**15**	94	0:30						
**16**	44	1:00						
**17**	72	2:00						
**18**	Go to step 9	12X						
**19**	72	7:00						
**20**	4	∞						

* *tp*TAIL-PCR, targeting probe thermal asymmetric interlaced PCR.
