# Supplementary material for: Inheritance of DNA Transferred from American Trypanosomes to Human Hosts
Source: PLoS One. 2010 Feb 12;5(2):e9181. doi: 10.1371/journal.pone.0009181 (PMC2820539; doi:10.1371/journal.pone.0009181)
Supplement: Table S3 — Transfer of minicircle sequences of kDNA from Trypanosoma cruzi to the human genome. (0.14 MB PDF) [file pone.0009181.s009.pdf]

Table S3. Transfer of Minicircle Sequences of kDNA from *Trypanosoma cruzi* to the Human Genome

A) Somatic Cells

Patient	EMBL	kDNA	Human DNA	Intermediate recombination site	kDNA *E-value*	Human chromosome	*Locus*	*E-value*
**1**	FM207254	1-325	310-360	GGAGATGCATAAATTT	$6.0e^{-29}$ emb|X04680.1	5	AC008804 (Not determinate)	$2.0e^{-14}$
**1**	FM207255	1-360	347-441	CCTCCCAAGACTAA	$7.0e^{-48}$ gb|M19188.1	X	AL732374 (LINE-1)	$1.0e^{-31}$
**1**	FM207256	1-256	244-574	CCCCTCCCAAAAC	$2.0e^{-29}$ gb|M19188.1	1	AL035414 (LINE-1)	$6.0e^{-120}$
**1**	FM207257	1-263	255-648	AATCGAACC	$7.0e^{-8}$ emb|AJ747941.1	10	AL136085 (LINE-1)	0.0
**2**	FM207258	1-602	588-727	AACCCCAATCGAACC	$2.0e^{-58}$ gb|M18814.1	9	AL354711 (LINE-1)	$2.0e^{-58}$
**2**	FM207259	54-479	1-53	AACCAATAACAGGC	$3.0e^{-41}$ gb|AY490898.1	11	AC090124 (LINE-1)	$2.0e^{-14}$
**2**	FM207260	1-597	586-1221	CAATCGAACCCC	$6.0e^{-38}$ gb|M19188.1	X	AL022578 (LINE-1)	0.0
**2**	FM207261	1-288/ 502-765	283-506	AACCAA/ TGGTT	$4.0e^{-9}$ gb|EU088397.1	X	AL732374 (LINE-1)	$9.0e^{-43}$
**2**	FM207262	1-281/ 729-1045	262-749	ACCAACCCCAATCGAACC/ ATCTCACCCGTACATTATT	$2.0e^{-7}$ gb|AF399842.1	12	AC084364 (LINE-2)	0.0
**2**	FM207263	1-654	642-1101	CCCAACCGAACCC	$2.0e^{-37}$ gb|AF399842.1	13. 7	AL136523 AC012596 (LINE-1)	$7.0e^{-97}$ $1.0e^{-49}$

**2**	FM207264	1-279/ 508-838	271-507	AATCGAACC/ GGGTTCGAT	$2.0e^{-13}$ gb|AF399842.1	14. 10	AL049875. AL591363 (LINE-1)	$1.0e^{-111}$ $4.0e^{-19}$
**2**	FM207265	1-286	275-393	CCCAATCGAACC	$4.0e^{-10}$ gb|EU088391.1	7	AC012596 (LINE-1)	$2.0e^{-47}$
**3**	FM207266	1-287	267-1134	ACACAACCCCAATCGAACC	$4.0e^{-10}$ gb|EU088391.1	5	AC108104 (LINE-1)	0.0
**3**	FM207277	1-286/ 479-773	275-496	CCCAATCGAACC/ GGTTCGATTGGGGTTGGT	$3.0e^{-11}$ gb|EU088397.1	7	AC012596 (LINE-1)	$9.0e^{-49}$
**3**	FM207268	1-287	277-633	CCAATCGAACC	$3.0e^{-11}$ gb|EU088391.1	22	Z82173 (Not determinate)	$1.0e^{-179}$
**3**	FM207269	1-286	270-1230	CCAACCCCCATCGAACC	$1.0e^{-11}$ gb|EU088395.1	7	AC073647 (LINE-1)	0.0
**3**	FM207270	1-295	278-974	ACCAACCCCAATCGAACC	$2.0e^{-9}$ gb|EU088399.1	2	AC007741 (MER)	0.0
**3**	FM207271	1-285	278-544	ATCGAACC	$4.0e^{-11}$ gb|EU088391.1	3	AC117454 (LINE-1)	$7.0e^{-106}$
**3**	FM207272	1016-1301	1-1031	GGTTCGATTGGGGTTG	$4.0e^{-10}$ gb|AF242562.1	4	AC108038 (LINE-1)	0.0
**3**	FM207273	1-266/ 1381-1666	259-1389	AT CGAACC/ GGTTCGATT	$1.0e^{-9}$ gb|EU088401.1	X. 10	AL442646 AL591363 (LINE-1)	0.0 $5.0e^{-20}$
**4**	FM207274	252-366	1-257/ 353-473	ACACCAA/ CCCTCCCAAAACCA	$9.0e^{-33}$ gb|AF399842.1	X. 8	AL732374 AC020783 (LINE-1)	$1.0e^{-37}$
**5**	FM207275	47-338	1-60	TGAACGCCCCTCCCAA	$4.0e^{-10}$ gb|EU088392.1	1	AL359742 (PARP-1 gene)	$1.0e^{-12}$
**5**	FM207276	368-657	1-380	GACCCCCCCTCC	$6.0e^{-8}$ gb|EU088392.1	X	AL035246 (LINE-1)	0.0

**5**	FM207277	93-369	1-110	GACCGCCCCTCCCAAAAC	$4.0e^{-10}$ gb|EU088387.1	7	NG_007092 (CNTNAP2 gene)	$4.0e^{-38}$
**5**	FM207278	192-471	1-194	GAC	$5.0e^{-9}$ gb|EU088401.1	3	AC099047 (LINE-1)	$9.0e^{-92}$
**6**	FM207279	1-78	62-528	CCCCAATCGAACCCCA	$9.0e^{-11}$ gb|EU088400.1	2	AC103564 (CpG island)	0.0
**6**	FM207280	1-265	262-502	AACC	$3.0e^{-37}$ gb|AF399842.1	X	AL732374 (LINE-1)	$4.0e^{-90}$
**6**	FM207281	1-579/ 723-1041	566-722	ACCCCAATCGAACC	$4.0e^{-40}$ gb|AF399842.1	8	AC219164 (LINE-1)	$8.0e^{-65}$
**6**	FM207282	284-704	1-292	ATACACC	$1.0e^{-39}$ gb|AF399842.1	10	AL392108 (LINE-1)	$6.0e^{-102}$
**6**	FM207283	1-288/ 540-654	285-545	AACC/ TACACC	$9.0e^{-36}$ gb|U07846.1	X	AL732374 (LINE-1)	$9.0e^{-68}$
**7**	FM207284	1-205/ 459-752	206-463	TTTGA	$3.0e^{-11}$ gb|EU088397.1	6	AL590035 (LINE-2)	$6.0e^{-128}$
**7**	FM207285	1-290	278-823	GGGGGTTCATTC	$7.0e^{-39}$ gb|AF399842.1	4	AC118562 (LINE-1)	0.0
**7**	FM207286	1-297	278-744	TGGGAGGGGGGGTCAAATG	$2.0e^{-8}$ gb|AF002202.1	13	AL157363 (LINE-1)	0.0
**7**	FM207287	1-427	422-1109	GGTCAA	$1.0e^{-38}$ gb|U07846.1	4	AC105444 (LINE-1)	0.0
**8**	FM207288	1-289	269-560	ACACCAACCCCCAATCGAACC	$2.0e^{-8}$ gb|U07845.1	10	AL392043 (Not determinate)	$4.0e^{-135}$
**9**	FM207289	1-414	406-646	AATCGAACC	$5.0e^{-36}$ gb|AF399842.1	14	AL049875 (LINE-1)	$1.0e^{-112}$
**9**	FM207290	1-280	277-384	AACC	$5.0e^{-9}$ gb|EU088401.1	5	AC114982 (LINE-1)	$5.0e^{-53}$

**10**	FM207291	145-307	1-150	TACACC	$1.0e^{-33}$ gb|AF399842.1	X	AL732374 (LINE-1)	$6.0e^{-53}$
**10**	FM207292	1-267	246-377	ACCAACCCCAATCGAACCAACC	$1.0e^{-10}$ gb|EU088396.1	12	AC084364 (Alu)	$1.0e^{-43}$
**13**	FM207293	1-263	256-311	ATCGAACC	$1.0e^{-10}$ gb|EU088396.1	10	AL591363 (Not determinate)	$4.0e^{-17}$
**14**	FM207294	1-261	248-346	ACCCAATCGAACC	$3.0e^{-11}$ gb|EU088389.1	1	AL606535 (Not determinate)	$3.0e^{-32}$
**14**	FM207295	158-360	1-160/ 351-576	TGA/ ACCCAAAACC	$2.0e^{-26}$ gb|EU088379.1	18. 4	AP001847 (LINE-1) AC097512 (Alu)	$3.0e^{-61}$ $1.0e^{-89}$
**15**	FM207296	1-636	634-713	CCC	$1.0e^{-33}$ gb|AF399842.1	1	AC096540 (Not determinate)	$3.0e^{-17}$
**15**	FM207297	1-258	241-509	CCAACCCCAATCGAACC	$7.0e^{-32}$ gb|AF399842.1	1. X	AC096540 (Not determinate) AL732374 (LINE-1)	$2.0e^{-15}$ $2.0e^{-57}$
**15**	FM207298	1-596	584-709	ATTACACCAACCCCAA	$1.0e^{-32}$ gb|AF399842.1	5	AC003998 (Alu)	$3.0e^{-46}$
**15**	FM207299	1-306	293-443	CCCCAATCGAACC	$1.0e^{-30}$ gb|AF399842.1	1. X	BK004196 (OR1-17 gene) AL732374 (LINE-1)	$7.0e^{-17}$ $2.0e^{-30}$
**15**	FM207300	1-91	85-488	TACACC	$4.0e^{-18}$ gb|AF399842.1	X	AL732374 (LINE-1)	$2.0e^{-88}$
**16**	FM207301	1-360	351-578	CCAAGACTAA	$1.0e^{-36}$ gb|AF399842.1	X	AL732374 (LINE-1)	$2.0e^{-57}$

**16**	FM207302	55-199/ 597-915	1-54/ 188-599	CCCCTCCCAAAA/ TGA	$3.0e^{-31}$ gb|AY485269.1	X	AL732374 (LINE-1)	$2.0e^{-110}$
**16**	FM207303	223-540	1-228	GACGGC	$9.0e^{-13}$ gb|AF399842.1	4	AC108869 (LINE-1)	$2.0e^{-107}$
**18**	FM207304	1-283	280-370	AACC	$7.0e^{-147}$ gb|EU088379.1	8	AC104006 (LINE-1)	$1.0e^{-24}$
**18**	FM207305	1-285	283-425	ACC	$1.0e^{-11}$ gb|EU088394.1	X	AL732374 (LINE-1)	$1.0e^{-16}$
**18**	FM207306	1-302/ 499-628	303-513/ 614-767	TCCTCCTGGTTTAGT/ GATTGGGGTTGGTGT	$1.0e^{-141}$ gb|EU088388.1	X	AL732374 (LINE-1)	$1.0e^{-48}$
**19**	FM207307	1-244/ 680-733	227-683	ACCAACCCCAATCGAACC/ GGAG	$8.0e^{-18}$ gb|EU088396.1	7	AC002433 (Not determinate)	0.0
**19**	FM207308	1-275	255-593	CACACCAACCCCAATCGAACC	$4.0e^{-10}$ gb|EU088401.1	2	AC010987 (Not determinate)	$3.0e^{-161}$
**19**	FM207309	1-286	275-419	CCCAATCGAACC	$4.0e^{-10}$ gb|EU088391.1	7	AC006381 (ERV)	$2.0e^{-53}$
**19**	FM207310	1-275	256-511	ACACCAACCCCAATCGAACC	$1.0e^{-11}$ gb|EU088401.1	9	AL162732 (LINE-2)	$5.0e^{-63}$
**19**	FM207311	1-293	276-919	CCAATCGAACCTCATCTT	$4.0e^{-10}$ gb|EU088391.1	2	AC009225 (ADAM 23 gene)	0.0
**19**	FM207312	1-456	434-571	CATACACCAACCCCAATCGAACC	$5.0e^{-31}$ gb|U07846.1	3	AC112214 (Not determinate)	$2.0e^{-49}$
**19**	FM207313	1-283	280-403	AACC	$3.0e^{-144}$ gb|EU088392.1	X	AL732374 (LINE-1)	$3.0e^{-14}$
**19**	FM207314	1-283	250-445	TTCCCATCTATATTACCCAACCCCAA TCGAACC	$1.0e^{-144}$ gb|EU088392.1	X	AL732374 (LINE-1)	$3.0e^{-27}$

**19**	FM207315	1-344	343-506	CC	9.0e-121 gb|EU088388.1	X	AL732374 (LINE-1)	1.0e-52
**19**	FM207316	1-418	410-843	AATCGAACC/ AACC	5.0e-36 gb|AF399842.1	14. X	AL049875 AL732374 (LINE-1)	1.0e-85 4.0e-27
**22**	FM207317	140-257	1-145/ 243-534	TACACC/ CCCTCCCAAGACTAA	1.0e-38 gb|AF399842.1	X	AL732374 (LINE-1)	2.0e-56
**24**	FM207318	675-1078	1-684	GCATCTCCCCC	5.0e-24 gb|AY490898.1	16	AC109597 (Not determinate)	0.0
**24**	FM207319	1-323	303-1268	ACACCAACCCCAATCGAACC	4.0e-24 gb|EU088393.1	7	AC073647 (LINE-2)	0.0
**24**	FM207320	347-645	1-349	TGA	5.0e-9 gb|EU088401.1	19	AC063977 (Alu)	1.0e-173
**25**	FM207321	1-220	207-563	ACCCCAATCGAACC	2.0e-32 gb|AF399842.1	X	AL732374 (LINE-1)	2.0e-56
**25**	FM207322	1-435	430-528	AATTT	3.0e-26 gb|AF399842.1	11	AC025300 (LINE-1)	5.0e-22
**26**	FM207323	1-325	313-404	CCCTCCCAAGAAC	9.0e-7 gb|AY490903.1	X	AL732374 (LINE-1)	3.0e-26
**26**	FM207324	50-352	1-57	GGGAGGG	2.0e-15 gb|AY490903.1	1	AL354890 (Not determinate)	3.0e-19
**26**	FM207325	98-486	1-105	ACACCAAC	1.0e-31 gb|U07846.1	7	AC022261 (LINE-1)	4.0e-26
**26**	FM207326	1-276	273-552	TTCA	1.0e-11 gb|EU088401.1	6	AL355522 (CLIC5 gene)	2.0e-137
**26**	FM207327	1-660	649-767	CCCAATCGAACC	1.0e-34 gb|AF399842.1	7	AC012596 (LINE-1)	4.0e-47
**27**	FM207328	1-324	310-500	GAGATGCATAAATTT	1.0e-30 gb|AF399842.1	X	AL732374 (LINE-1)	2.0e-62
**27**	FM207329	1-286	284-570	TCA	1.0e-11	8	AC074252	8.0e-137

					gb|EU088397.1		(LINE-1)	
**27**	FM207330	1-479	478-521	GC	$7.0e^{-48}$ gb|AY490904.1	1	AL590101 (LINE-1)	$2.0e^{-10}$
**28**	FM207331	108-268	1-113	GCATCT	$3.0e^{-20}$ gb|EU088400.1	15	DQ232883 (TRIM69 gene)	$2.0e^{-45}$
**28**	FM207332	83-200	1-97/ 195-401	TTAGTCTTGGGAGG/ GGTGTA	$3.0e^{-33}$ gb|AF399842.1	X	AL732374 (LINE-1)	$1.0e^{-57}$
**28**	FM207333	1-106	100-578	TGGTGTA	$3.0e^{-38}$ gb|AF399842.1	X	AL732374 (LINE-1)	$9.0e^{-55}$
**29**	FM207334	1-267/ 371-452	246-370	ACCAACCCCAATCGAACCAACC	$9.0e^{-21}$ gb|AY490898.1	12	AC084364 (Alu)	$2.0e^{-43}$
**29**	FM207335	1-387	375-494	CCCAATCGAACCC	$8.0e^{-21}$ gb|EU088393.1	7	AC012596 (LINE-1)	$2.0e^{-48}$
**29**	FM207336	161-451	1-177	GACCCCCCCTCCCAAAA	$4.0e^{-10}$ gb|EU088388.1	14	AL049776 (LINE-1)	$1.0e^{-76}$
**29**	FM207337	166-445	1-187	GACGGCCCCTCC	$2.0e^{-7}$ gb|EU088399.1	X	AC142497 (MER)	$1.0e^{-77}$
**30**	FM207338	1-150/ 290-445	132-293	CTCCCAAGACTAAACCAGA/ GGTT	$3.0e^{-20}$ gb|AY490897.1	X. 8	AL732374 AC104006 (LINE-1)	$1.0e^{-16}$ $1.0e^{-20}$
**30**	FM207339	1-246	241-728	CGAACC	$2.0e^{-31}$ gb|U07846.1	1. X	BK004196 (OR1-17 gene) AL732374 (LINE-1)	$2.0e^{-16}$ $1.0e^{-54}$
**31**	FM207340	1-296	289-566	ATCGAACC	$1.0e^{-10}$ gb|EU088385.1	11. 20	AP002345 RP3-322G13 (Not determinate)	$3.0e^{-90}$ $3.0e^{-33}$
**31**	FM207341	1-80/ 707-822	72-712	CCCTCCCA/ TACACC	$3.0e^{-36}$ gb|AF399842.1	X	AL732374 (LINE-1)	$7.0e^{-58}$

**31**	FM207342	125-246/ 570-692	1-130/ 243-574/ 675-909	ATAC/ AAAC/ ACACC/ CCCTCCCAAGACTAAACC	$1.0e^{-41}$ gb|AF399842.1	X	AL732374 (LINE-1)	$2.0e^{-58}$
**33**	FM207343	1-264 422-483	260-421 468-513	AACCA/ CAACCCCAATCGAACC	$1.0e^{-10}$ gb|EU088396.1	X.1	AL732374 (LINE-1) AC096540.2 (Not determinate)	$1.0e^{-33}$ $6.0e^{-09}$
**34**	FM207344	1-427	412-517	GCCCCTCCCAAAACC	$5.0e^{-24}$ gb|U07846.1	3	AC092059 (LINE-2)	$1.0e^{-33}$
**36**	FM207345	1-287	259-373	TGAAGGCCCCTCCCA AAAC	$2.0e^{-13}$ gb|AF242562.1	3	AC092059 (LINE-2)	$8.0e^{-34}$
**36**	FM207346	1-304	296-732	GGGAGGGGC	$1.0e^{-11}$ gb|EU088385.1	12	ABC10-45521700B16 (LINE-1)	0.0
**36**	FM207347	264-315/ 1646-1933	1-276/ 307-1657	GAACGCCCCTCCC/ TAATTAACCTGT	$1.0e^{-10}$ gb|EU088393.1	7. 2	AC004988 (Alu) BC001197.1 (Theta 14-3-3 gene)	$1.0e^{-126}$ 0.0
**37**	FM207348	1-370	357-520	CCCTCCCAAGACTA	$7.0e^{-28}$ gb|U07846.1	X	AL732374 (LINE-1)	$6.0e^{-50}$
**37**	FM207349	1-376	362-454	CCCTCCCAAGACTAA	$6.0e^{-35}$ gb|AF399842.1	X	AL732374 (LINE-1)	$3.0e^{-34}$
**39**	FM207350	358-475	1-357/ 461-663	CCCTCCCAACTAA	$3.0e^{-32}$ gb|AF399842.1	X	AL732374 (LINE-1)	$9.0e^{-55}$
**39**	FM207351	1-356/ 590-711	346-605/ 701-1177	CCCTCCCAAGA/ CTCTGAATACACCAA	$2.0e^{-39}$ gb|AF399842.1	15	AC012378 (LINE-1)	0.0
**41**	FM207352	1-388	370-533	CCCCTCCCAAGACTAAACC	$5.0e^{-36}$ gb|AF399842.1	X	AL732374 (LINE-1)	$1.0e^{-52}$

**41**	FM207353	1-391	373-466	CCCCTCCCAAGACTAAACC	$1.0e^{-37}$ gb|AF399842.1	X	AL732374 (LINE-1)	$3.0e^{-34}$
**41**	FM207354	1-306	291-1153	CAACCCCAATCGAACC	$8.0e^{-20}$ gb|EU088393.1	5	AC108104 (LINE-1)	0.0
**42**	FM207355	1-136	126-395	CCAATCGAACC	$8.0e^{-22}$ gb|AY490903.1	Y	AC006370 (LINE-1)	$4.0e^{-133}$
**42**	FM207356	1-409	401-827	AATCGAACC	$6.0e^{-29}$ gb|U07846.1	14	AL049875 (LINE-1)	$2.0e^{-101}$
**44**	FM207357	137-241<br>432-580	1-142<br>242-439	GACCCC/ GACCGCCC	$7.0e^{-41}$ gb|AF399842.1	22. 5	AL022327 (Not determinate)<br>NM_015288 (PHD finger protein 15 gene)	$2.0e^{-59}$<br>$3.0e^{-93}$
**46**	FM207358	1-150<br>378-594	142-377<br>581-682	AATCGAACC/ ACCCCAATCGAACC	$5.0e^{-19}$ gb|EU088379.1	14. 5	AL049875<br>AC020980.6 (LINE-1)	$1.0e^{-111}$<br>$5.0e^{-14}$
**46**	FM207359	70-504	1-69<br>511-572	AACC	$9.0e^{-36}$ gb|AF399842.1	4. X	AC107213 (Not determinate)<br>AL390879 (LINE-1)	$7.0e^{-18}$<br>$2.0e^{-20}$
**46**	FM207360	1-382	379-438	AACC	$5.0e^{-37}$ gb|AF399842.1	8	AC104006 (LINE-1)	$2.0e^{-22}$
**48**	FM207361	1-360	348-572	CTCGCAAGACTAA	$9.0e^{-27}$ gb|AF399842.1	X	AL732374 (LINE-1)	$2.0e^{-57}$
**50**	FM207362	1-642	623-749	ACACCAACCCCAATCGAA CC	$3.0e^{-33}$ gb|AF399842.1	7	AC012596 (LINE-1)	$2.0e^{-47}$
**51**	FM207363	1-286	274-410	CCCAATCGAACCC	$2.0e^{-14}$ gb|EU088391.1	7	AC006381 (ERV1)	$3.0e^{-58}$
**51**	FM207364	1-285	266-350	ACACCAACCCCAATCGAACC	$8.0e^{-13}$ gb|EU088391.1	10	AL591363 (Not	$6.0e^{-19}$

							determinate)	
**51**	FM207365	1-283	276-368	ATCGAACC	$2.0e^{-14}$ gb|EU088391.1	1	AL606535 (MaLR)	$3.0e^{-32}$
**51**	FM207366	1-353/ 504-786	344-508	CCCTCCCCAA/ GGTT	$5.0e^{-141}$ gb|EU088396.1	X	AL732374 (LINE-1)	$2.0e^{-31}$
**55**	FM207367	1-360	346-446	CCCTCCCAAGACTAA	$7.0e^{-40}$ gb|AF399842.1	X	AL732374 (LINE-1)	$1.0e^{-31}$
**55**	FM207368	1-325	311-562	GAGATGCATAAATTT	$1.0e^{-30}$ gb|AF399842.1	X	AL732374 (LINE-1)	$2.0e^{-62}$
**56**	FM207369	1-285	270-341	CAACCCCAATCGAACC	$1.0e^{-10}$ gb|EU088392.1	1	BK004196 (OR1-17 gene)	$2.0e^{-20}$
**56**	FM207370	1-360	346-438	CCCTCCCAAGACTAA	$7.0e^{-40}$ gb|AF399842.1	X	AL732374 (LINE-1)	$6.0e^{-34}$
**58**	FM207371	1-41/ 86-470	38-97	CGGT/ GTAGGGTTCGAT	$6.0e^{-35}$ gb|AF399842.1	10	AL591363 (MER)	$5.0e^{-18}$
**58**	FM207372	1-356	353-520	AACC	$7.0e^{-34}$ gb|AF399842.1	X	AL732374 (LINE-1)	$2.0e^{-23}$
**58**	FM207373	1-76 221-310 413-585	67-231 308-424	CCCTCCCAA/ TGATGTTGGTT/ GGA/ GGTTCGATTGGG	$2.0e^{-17}$ gb|AY490897.1	X. 7	AL732374 AC012596 (LINE-1)	$1.0e^{-33}$ $4.0e^{-46}$
**59**	FM207374	1-285/ 371-535	268-374	ACCAACCCCAATCGAACC/ TGGG	$8.0e^{-23}$ gb|AY490897.1	12	AC084364 (Alu)	$8.0e^{-42}$
**59**	FM207375	1-267 362-392	243-361	CACACCAACCCCAATCGAACCCAAC	$5.0e^{-9}$ gb|EU088396.1	4	AC106898 (MIR)	$3.0e^{-45}$
**59**	FM207376	184-296	1-185 303-395	CT/ CCCTCCCAA	$3.0e^{-33}$ gb|AF399842.1	X	AL732374 (LINE-1)	$2.0e^{-54}$
**60**	FM207377	1041-1295	1-1047/ 1289-	TTGGGAG/ TGGTGTA	$6.0e^{-33}$ gb|AF399842.1	6. X	AL356579 (Alu)	0.0 $5.0e^{-42}$

			1403				AL732374 (LINE-1)	
**60**	FM207378	1-293 577-748	294-585	ACACCAACCC	$2.0e^{-31}$ gb|AF399842.1	X	AL732374 (LINE-1)	$3.0e^{-61}$
**61**	FM207379	1-303	291-492	CCCCAATCGAACC	$9.0e^{-13}$ gb|AF242560.1	5	AC008581 (Not determinate)	$2.0e^{-87}$
**61**	FM207380	529-820	1-555	GAAGCCCCCTCCCAAAACCACACTTTC	$6.0e^{-8}$ gb|EU088393.1	5	AC093531 (Not determinate)	0.0
**63**	FM207381	281-475	1-310 465-724	ACAGTCCCCA/ CCAATCGAACC	$9.0e^{-23}$ gb|AF399841.1	10. Y	AC005871 (citb-109 gene) AC006370 (LINE-1)	$2.0e^{-136}$ $8.0e^{-121}$
**63**	FM207382	1-331	321-511	CCAATCGAACC	$4.0e^{-11}$ gb|AY490905.1	11	AC025972 (MER)	$2.0e^{-88}$
**65**	FM207383	1-137/ 281-348	129-293	CCCTCCCAA/ TTGATGTTGGTTC	$2.0e^{-16}$ gb|EU088389.1	X	AL732374 (LINE-1)	$7.0e^{-34}$
**66**	FM207384	1-529	526-777	CACA	$4.0e^{-83}$ gb|AY490904.1	6	AL357513 (COX6A1 pseudogene)	$2.0e^{-90}$
**67**	FM207385	1-291	279-415	CCCAATCGAACCC	$1.0e^{-10}$ gb|AY490894.1	7	AC006381 (Not determinate)	$4.0e^{-57}$
**67**	FM207386	1-274	263-312	CCAATCGAACCC	$2.0e^{-13}$ gb|AF242563.1	7	AC005999 (MIR)	$2.0e^{-15}$
**67**	FM207387	1-281	267-723	CCCCAATCGAACCCC	$1.0e^{-9}$ gb|EU088400.1	9	AL162252 (LINE-1)	0.0
**69**	FM207388	1-292	272-470	ACACCAACCCCAATCGAACCC	$1.0e^{-10}$ gb|U07845.1	18	AC044873 (Family (CA)n)	$2.0e^{-60}$

**69**	FM207389	1-303	295-680	AATCGAACC	$8.0e^{-39}$ gb|AF399842.1	6	DQ249181 (haplotype HLA)	0.0
**71**	FM207390	1-286	267-839	CACCAACCCCAATCGAACC	$3.0e^{-11}$ gb|U07846.1	6	AL031121 (LINE-1)	0.0
**71**	FM207391	1-271	254-815	CCAACCCCAATCGAACC	$1.0e^{-11}$ gb|EU088388.1	10	AL138772 (LINE-1)	0.0
**71**	FM207392	1-304	292-757	CCCCAATCGAACC	$1.0e^{-10}$ gb|EU088401.1	14	AL929600 (Alu)	0.0
**71**	FM207393	1-326	325-516	CA	$1.0e^{-11}$ gb|EU088401.1	11	AC009869 (LINE-1)	$5.0e^{-63}$
**74**	FM207394	1-143	130-397	AGCCCCAATCGAAC	$9.0e^{-15}$ gb|EU088401.1	1. X	BK004196.1 (OR1-17 gene) AL732374 (LINE-1)	$2.0e^{-20}$ $7.0e^{-54}$
**75**	FM207395	79-148	1-84 149-413	TACACC	$3.0e^{-29}$ gb|AF399842.1	5. 9	AC024586 (Not determinate) AL590397 (Tyrosine phosphatase gene)	$2.0e^{-06}$ $5.0e^{-132}$
**78**	FM207396	1-284/ 344-395	277-350	ATCGAACC/ CCGGAGA	$2.0e^{-8}$ gb|EU088393.1	10	AL591363 (Not determinate)	$4.0e^{-19}$
**79**	FM207397	1-183 375-401	172-374 394-670	CCCTCCCAAGAC/ ATCGAACC	$1.0e^{-39}$ gb|AF399842.1	X. 8	AL732374 KB1967D12 (LINE-1)	$4.0e^{-54}$ $5.0e^{-129}$
**79**	FM207398	1-436/ 538-591	419-541	ACCAACCCCAATCGAACC/ GGAG	$2.0e^{-42}$ gb|AF399842.1	12	AC084364 (Alu)	$3.0e^{-41}$
**81**	FM207399	1-72 181-	61-180 199-266	CCAAATCGAACC/ CCCTAATCGAACC	$9.0e^{-5}$ gb|EU088394.1	7.1	AC012596 (LINE-1)	$1.0e^{-48}$ $6.0e^{-14}$

		311					BK004196.1 (OR1-17 gene)	
**81**	FM207400	1-77	68-302	CCCTCCCAAG	$4.0e^{-22}$ gb|AY490903.1	X	AL732374 (LINE-1)	$5.0e^{-54}$
**82**	FM207401	1-154	140-208	CCAATCGAACCACCA	$2.0e^{-17}$ gb|AF399842.1	1	BK004196.1 (OR1-17 gene)	$3.0e^{-09}$
**82**	FM207402	1-153	151-701	ACC	$8.0e^{-16}$ gb|EU088396.1	8	AP004293 (Alu)	0.0
**82**	FM207403	196-422	1-195 410-478	CCCCAATCGAACC	$2.0e^{-36}$ gb|AF399842.1|	X. 1	AL031319 (Not determinate) BK004196.1 (OR1-17 gene)	$3.0e^{-97}$ $1.0e^{-18}$
**83**	FM207404	145-207	1-152	TACACCAA	$6.0e^{-6}$ gb|AY490900.1	5	AC114982 (LINE-1)	$6.0e^{-51}$
**85**	FM207405	1-171	165-215	CCTCCCAAGACTAAACC	$7.0e^{-30}$ gb|DQ241812.1	7	AC018647 (LINE-1)	$2.0e^{-19}$
**85**	FM207406	1-133 256-404	130-262 402-729	AACC/ TACACCAA/ TCT	$2.0e^{-36}$ gb|AF399842.1	7. X	AC003073 AL732374 (LINE-1)	$1.0e^{-53}$ $1.0e^{-50}$
**85**	FM207407	1-130 243-419	116-242 380-492	AACCCCAATCGAACCACCA/ CCTTATATTACACCAACCCCAATCGAACC	$1.0e^{-32}$ gb|AF399842.1	9. 5	AL583827 AC113414 (LINE-1)	$2.0e^{-49}$ $2.0e^{-34}$
**86**	FM207408	1-380	369-532	CCCTCCCAAGAC	$2.0e^{-42}$ gb|AF399842.1	X	AL732374 (LINE-1)	$1.0e^{-51}$
**86**	FM207409	219-493	1-221	TGA	$1.0e^{-9}$ gb|EU088391.1	4	AC113152 (LINE-1)	$2.0e^{-87}$
**86**	FM207410	556-834	1-558	GAC	$4.0e^{-10}$ gb|EU088391.1	2	AC068616 (LINE-1)	0.0

B) Germline cells

Patient	EMBL	kDNA	Human DNA	Intermediate recombination site	kDNA *E-value*	Human chromosome	*Locus*	*E-value*
**4**	FM207148	134-196/<br>234-254/<br>377-397/<br>520-637	1-146/<br>196-241/<br>250-384/<br>394-527	TACACCAACCCAA/<br>AACC/<br>TACACCAA	8e-36<br>(gb|AF399842)	7<br>X	AF104455.1<br>(LINE-1)<br>AL732374.14<br>(LINE-1)	$8e^{-50}$<br>$7e^{-45}$
**4**	FM207149	1-382/<br>506-623	368-511/<br>608-871	CCCTCCCAAGACTAA/<br>TACTCC/<br>ACCCTCCCAAGACTAA	2e-38<br>(gb|AF399842)	X	AL732374.14<br>(LINE-1)	$1e^{-49}$
**4**	FM207150	86-176/<br>275-420/<br>796-937/<br>1271-<br>1297	1-101/<br>177-282/<br>421-800/<br>926-1270	TTGACCCCCCCTCCCA/<br>TGACGGCCCCTCCCAAAA/<br>TGAA/<br>CCCTCCCAAAAC	2e-23<br>(gb|AY490898)	13<br>16<br>2<br>X	AL137140.12<br>(CpG island)<br>AC125796.2<br>(Not determinate)<br>AC093690.5<br>(Not determinate)<br>AL732374.14<br>(LINE-1)	$4e^{-32}$<br>$5e^{-43}$<br>$4e^{-171}$<br>$5e^{-56}$
**4**	FM207151	1-62/<br>264-283/<br>414-782	57-263/<br>280-420/<br>777-848	TGGTGT/ GTGT/ TTGGGAG	8e-82<br>(gb|AY490904)	X	AL732374.14<br>(LINE-1)	$1e^{-60}$
**4**	FM207152	35-333	1-42/<br>327-798	TACACCAA/<br>CTACACCA/<br>GAGATGC	5e-52<br>(gb|AF399842)	8<br>12	BK000383.1<br>(LINE-1)<br>AC084364.20<br>(L2 e Alu)	$8e^{-12}$<br>0.0
**4**	FM207153	1-249/<br>344-445	232-349	CCCTCCCAAGACTAAACC/<br>ACACCA	1e-44<br>(gb|AF399842)	X	AL732374.14<br>(LINE-1)	$5e^{-44}$
**4**	FM207154	104-819	1-114/<br>808-1138	TACACCAA/<br>GGGTGAGATGC	1e-56<br>(gb|AF399842)	X<br>12	AL732374.14<br>(LINE-1)<br>AC084364.20<br>(L2 e Alu)	$1e^{-35}$<br>$5e^{-156}$
**4**	FM2071455	1-968	958-1287	GGGTGAGATGC	6e-54	12	AC084364.20<br>(L2 e Alu)	$6e^{-156}$

					(gb|AF399842)			
**4**	FM207156	1-699	683-1353	TTTTGGGAGGGGGGTTC	2e-23 (gb|AY490898)	8	AC016868.8 (MER31A)	0.0
**4**	FM207157	108-581/ 634-739	1-113/ 566-638	ACACCA/ CCCTCCCAAGACTAAA/ ACACC	1e-53 (gb|AF399842)	X	AL732374.14 (LINE-1)	$2e^{-25}$
**4**	FM207158	130-440	1-142	T TAGTCTTGGG AG	4e-38 (gb|AF399842)	X	AL732374.14 (LINE-1)	$8e^{-48}$
**6**	FM207159	1-287	265-930	ATTACA CCAACCCCAA TCGAACC	1e-10 (gb|EU088393)	12	AC125629.1 (LINE-1)	0.0
**6**	FM207160	1-208	193-481	ATGGTTTTGGGAGGGG	2e-30 (gb|AF399842)	6	AL160399.13 (Not determinate)	$2e^{-132}$
**6**	FM207161	1-356/ 473-591	341-472	CCCTCCCAAG ACTAAA	9e-54 (gb|AF399842)	X	AL732374.14 (LINE-1)	$9e^{-49}$
**6**	FM207162	1-198	191-1176	ACTAAACC	1e-36 (gb|AF399842)	X	AL135784.4 (LINE-1) AL732374.14 (LINE-1)	0.0 $4e^{-20}$
**6**	FM207163	192-360	1-198/354-1285	ACACCAA/ CCCTCCC	1e-37 (gb|AF399842)	5 8	AC129713.1 (Not determinate) AC009902.13 (LINE-1)	$1e^{-87}$ 0.0
**10**	FM207164	1-280	264-336	CCAACCCCAATCGAACC	6e-09 (gb|EU088391)	1	AC096540.2 (Not determinate)	$2e^{-18}$
**10**	FM207165	93-173	1-109	ACACAGCTCTACACCAA	9e-16 (gb|AF399842)	X	AL359973.11 (LINE-1)	$2e^{-37}$
**25**	FM207166	205-314	1-210	TACACC	8e-39 (gb|AF399842)	X	AL732374.14 (LINE-1)	$2e^{-59}$
**25**	FM207167	203-469/508-574/614-793	1-212/ 470-507/ 575-613 775-905	TGGTTTTG GG ATGGTTTTGGGAGGGG CGG	9e-25 (gb|AY490898)	11 16	NM_002457.2 (Mucin gene) AC092145.2 (Not determinate)	$2e^{-78}$ $4e^{-49}$

**25**	FM207168	1-252	253-485	Not found	3e-21<br>(gb|AY490898)	11	M22404.1<br>(Mucin gene)	$3e^{-59}$
**25**	FM207169	1-332	322-592	GGGGGAGATGC	1e-21<br>(gb|U07846)	11	AC107948.7<br>(Not determinate)	$2e^{-126}$
**25**	FM207170	1-309	297-650	CAATCGAACCACC	3e-09<br>(gb|EU088396)	1	AL391883.17<br>(Not determinate)	$2e^{-164}$
**25**	FM207171	1-94/<br>122-142	83-<br>129/139-<br>246	CCCTCCCAAGAC/<br>TACACCAA/<br>AACC	3e-23<br>(gb|AY490897)	4	AC093747.2<br>(LINE-1)	$3e^{-44}$
**25**	FM207172	66-<br>554/1217-<br>1464	1-71/539-<br>1223	TACACC/<br>CCCTCCCAAGACTAAA/<br>TACA CCA	1e-63<br>(gb|AF399842)	X	AL732374.14<br>(LINE-1)	5e -<br>56
**27**	FM207173	107-<br>209/250-<br>270/917-<br>936	1-113/210-<br>249<br>250-<br>916/938-<br>1041	TACACCA<br>TACACCAACCCCAATCGAACC	2e-40<br>(gb|AF399842)	X<br>2	AL732374.14<br>(LINE-1)<br>AC116654.4<br>(LINE-1)	$1e^{-36}$<br>0.0
**27**	FM207174	107-215/<br>350-468/<br>494-514	1-111/<br>210-<br>358/454-<br>501/<br>513-1096	CCCTCC/ TACACCAA<br>CCCTCCCAAGACTAA	2e-40<br>(gb|AF399842)	X<br>X	AL732374.14<br>(LINE-1)<br>AL670463.3<br>(LINE-1)	$8e^{-36}$<br>0.0
**27**	FM207175	1-<br>115/264-<br>284<br>1117-<br>1221	108-<br>269/277-<br>1118<br>1215-1336	TTGGTGTA/ GGTTCG/ TTGGTGTA	2e-40<br>(AF399842)	1<br>X	AL513363.8<br>(LINE-1)<br>AL732374.14<br>(LINE-1)	0.0<br>$1e^{-47}$
**27**	FM207176	1-263	251-409	CCCCAATCGAACC	1e-11<br>(gb|EU088396)	1	AC119426.2<br>(Not determinate)	$4e^{-70}$
**27**	FM207177	1-287	271-472	CCAACCCCAATCGAACC	2e-10<br>(gb|EU088394)	6	AM393849.1<br>(TXLNB gene)	$1e^{-76}$
**27**	FM207178	1-263	252-394	CCCAATCGAACC	2e-10<br>(gb|EU088396)	7	AC006381<br>(ERV1)	6e-55
**27**	FM207179	327-716	1-334	TACACCAA/<br>AACC	6e-51<br>(gb|AF399842)	5	AC113414<br>(LINE-1)	$4e^{-62}$

**27**	FM207180	1-300	290-408	CCCAATCGAACC	5e-11 (gb|EU088388)	7	AC012596.4 (LINE-1)	$5e^{-50}$
**27**	FM207181	1-65/ 172-321	57-172/312-857	TGGTGTATT/ TTGGTGTATT	4e-35 (gb|AF399842)	5	AC129713.1 (LINE-1)	$6e^{-167}$
**28**	FM207182	57-173/ 226-252/ 583-747/ 796-912	1-56/158-225/ 233-582/735-806/901-964	CCA AACTTGAACCCCCTCCCAA/ ACCAACCCCAATCGAACC/ CCCTCCCAAGACT/ TACACATACAC/ ACCCCCTCCCAA	3e-62 (gb|AF399842)	4	AP002027.1 (LINE-1)	$1e^{-145}$
**28**	FM207183	1-68/415-553	56-418	CCCCAATCGAACC/ CCCA	7e-36 (gb|AF399842)	X	AL049734 (LINE-1)	$5e^{-178}$
**26**	FM207184	649-961	1-648	Not found	8e-51 (gb|AF399842)	1	AL138801.20 (Not determinate)	0.0
**28**	FM207185	738-960	1-737/952-1065	ATCGAACCC	6e-34 (gb|AF399842)	5<br>7	AC108104.2 (LINE-1)<br>AC012596.4 (LINE-1)	0.0<br>$7e^{-31}$
**28**	FM207186	1-339/446-702	327-445	CCCAATCGAACCC	2e-45 (gb|AF399842)	7	AC012596.4 (LINE-1)	$8e^{-50}$
**28**	FM207187	1-294	287-408	ACCAACCCCAATCGAACC	2e-10 gb|EU088391	3	AC112214.2 (Not determinate)	$8e^{-47}$
**28**	FM207188	1-256/502-631	241-501/620-872	CCCCAATCGAACCACC/ CCCAATCGAAC C	1e-17 (gb|AF399842)	X<br>1	AL732374.14 (LINE-1)<br>BK004196.1 (OR1-17 gene)	$2e^{-59}$<br>$7e^{-19}$
**28**	FM207189	1-180	170-439	CCAATCGAACC	3e-14 (gb|EU088396)	Y	AC006370.2 (LINE-1)	$5e^{-132}$
**28**	FM207190	1-263/1055-1482	247-1054/1474-1564	CCAACCCCAATCGAACC/ CCCTCCCAA	2e-36 (gb|AF399842)	8<br>X	AC015528.14 (LINE-1)<br>AL732374.14 (LINE-1)	0.0<br>$1e^{-28}$
**28**	FM207191	1-278	262-898	TTTGGGAGGGGGGTTCA	2e-07 (gb|EU088393)	6	AL135908.13 (LINE-1)	0.0

**33**	FM207192	68-179/282-393/ 566-586	1-73/171-288/ 285-572/ 579-686	TACACC/<br>CCCTCCCAA/<br>TACACCA/<br>ATCGAACC	7e-56<br>(gb|AF399842)	X	AL732374.14<br>(LINE-1)	$8e^{-50}$
**33**	FM207193	1-722/950-1168	714-949	AATCGAACC	5e-48<br>(gb|AF399842)	14	AL049875.2<br>(LINE-1)	$4e^{-113}$
**33**	FM207194	167-411	1-174/412-784	TGACGGCC	9e-24<br>(gb|AY490898)	14<br>11	AL583742.2<br>(LINE-1)<br>M22405.1<br>(Mucin gene)	$6e^{-78}$<br>$5e^{-85}$
**34**	FM207195	397-690	1-415	GGTTCGATTGGGGTTGGTG	2e-32<br>(gb|AF399842)	X<br>1	AL732374.14<br>(LINE-1)<br>AL359752.11<br>(Not determinate)	$7e^{-32}$<br>$3e^{-163}$
**34**	FM207196	1-294	283-421	CCCAATCGAACC	5e-36<br>(gb|AF399842)	X<br>1	AL732374.14<br>(LINE-1)<br>BK004196.1<br>(OR1-17 gene)	$8e^{-35}$<br>$6e^{-20}$
**34**	FM207197	1-294	272-549	ATTACACCAACCCCAATCGAACC	9e-35<br>(gb|AF399842)	5<br>X	AC106787.2<br>(Not determinate)<br>AL732374.14<br>(LINE-1)	$3e^{-48}$<br>$4e-^{52}$
**34**	FM207198	1-294	277-859	ACCAACCCCAATCGAACC	1e-34<br>(gb|AF399842)	14	AL591768.2<br>(LINE-1)	0.0
**34**	FM207199	1-730	717-999	CCCCAATCGAACC	2e-53<br>(gb|AF399842)	1<br>X	BK004196.1<br>(OR1-17 gene)<br>AL732374.14<br>(LINE-1)	$1e^{-18}$<br>$4e^{-54}$
**34**	FM207200	1-722	708-848	AACCCCAATCGAACC	1e-54<br>(gb|AF399842)	9	AL354711.24<br>(LINE-1)	$3e^{-57}$
**34**	FM207201	640-926	1-639/902-942	AACCCCAATTGAACC	1e-35<br>(gb|AF399842)	1<br>9	AL138801.20<br>(LINE-1)<br>AL354711.24<br>(LINE-1)	0.0<br>9e-58
**34**	FM207202	434-819/1028-1174	1-451/801-1036	GGTTCGATTGGGGTTGG T/<br>TGGGAGCTGTAGACCGGAG/<br>GGTTCGATT	1e-50<br>(gb|AF399842)	12<br>14	AC084364.20<br>(LINE-2 and Alu)<br>AL049875.2<br>(LINE-1)	0.0<br>$2e^{-111}$

**36**	FM207203	1-150/298-362	151-310	GGTTCGATTGGGG	3e-20 (gb|AY490897)	1	AC119426.2 (LINE-1)	$1e^{-69}$
**36**	FM207204	1-191	182-662	CAATCGAACC	2e-25 (gb|AY490897)	13	AL136439.19 (POLR1D Gene)	0.0
**44**	FM207205	153-275	1-158/276-382	TGACCC	2e-22 (gb|AY490898)	2<br>11	BN000002.1 (IL-1 receptor gene)<br>AC139749.4 (rpt_family="(TGG)n	$8e^{-67}$<br>$8e^{-16}$
**44**	FM207206	759-913	1-766	ACACCAA	2e-32 (gb|AF399842)	3	AC092185.3 (LINE-1)	0.0
**44**	FM207207	512-655	1-518	GACCCCCCCTCCCAAA	6e-19 (gb|EU088379)	3	AC090886.1 (LINE-1)	$1e^{-142}$
**44**	FM207208	1-212	202-732	AGGGGGGGTCA	2e-20 (gb|EU088379)	16	AC010543.9 (LINE-1)	0.0
**44**	FM207209	207-386	1-220	TGAACCCCCCTCCC	2e-15 (gb|AF399842)	10	EF444970.1 (LINE-1)	$1e^{-100}$
**46**	FM207210	78-440	1-99	TGACCCCCCCTCCCAAAACCAT	2e-22 (gb|AY490898)	4	AC095055.3 (LINE-1)	$4e^{-26}$
**46**	FM207211	1-303/450-511	240-463	ACTCACAACCAACACCACCCTCCACACCCCC CACTCCTTATATTACACCAACCCCAATCGAACC/ TAGTCTTGGGAGGG	7e-23 (gb|AY490897)	X	AL732374.14 (LINE-1)	$1e^{-34}$
**46**	FM207212	1-382	370-430	CCCCAATCGAACC	1e-25 (gb|EU088396)	1	AC119426.2 (Not determinate)	$8e^{-16}$
**46**	FM207213	1-60/184-219	57-190/215-671	AACC/ ACACCAA/ AAACA	6e-13 (gb|AF399842)	6	AL731683.12 (MTCO3 pseudogene)	0.0
**46**	FM207214	241-315	1-258	TGAACCCCCCTCCCAAAA	2e-05 (gb|AF242562)	14	AC007955.4 (Not determinate)	$3e^{-120}$
**46**	FM207215	48-264/388-505/815-932	1-52/252-391/493-821/920-1061	TACACC/ CTCCCAAG ACTAA	2e-40 (gb|AF399842)	X	AL732374.14 (LINE-1)	$2e^{-53}$
**51**	FM207216	1-294	291-452	AACC	8e-09	X	AL732374.14	$1e^{-32}$

					(gb|U07846.1)		(LINE-1)	
**51**	FM207217	1-278	275-344	CCCCA ATCGAACC	4e-62 (gb|AY490894)	1	BK004196.1 (Gene OR1-17)	$3e^{-19}$
**51**	FM207218	1-278	275-438	AACC	3e-07 (gb|EU088401)	x	AL732374.14 (LINE-1)	$5e^{-31}$
**51**	FM207219	1-283	280-321	AACC	1e-10 (gb|EU088397)	12	AC120104 (LINE-1)	$1e^{-04}$
**51**	FM207220	1-387	373-465	CCCTCCCA AGACTAA	1e-44 (gb|AF399842)	x	AL732374.14 (LINE-1)	$3e^{-34}$
**51**	FM207221	142-424	1-147	TGAACG	6e-10 (gb|EU088401)	1	AL445426.20 (LINE-1)	$1e^{-62}$
**51**	FM207222	381-667	1-387	AAGTTGA	2e-13 (gb|EU088401)	19	AC136499.2 (LINE-1)	$2e^{-107}$
**51**	FM207223	40-323	1-55	TGAC CCCCCCTCCCAA	1e-11 (gb|EU088401)	1	AL359742.15 (ADPRT gene)	$8e^{-14}$
**67**	FM207224	220-479	1-230	GAAGCCCCCTC	3e-08 (gb|EU088397)	15	AC068722.6 (LINE-1)	$1e^{-108}$
**67**	FM207225	297-574	1-315	GAACGCCCCTCCCA AAACC	6e-12 (gb|EU088387)	9	AL354704.17 (LINE-1)	$7e^{-146}$
**67**	FM207226	83-370	1-104	TGATGAAC GCCCCTCCCA AAAC	4e-12 (gb|EU088395)	3	AC092059.2 (Not determinate)	$3e^{-32}$
**67**	FM207227	389-665	1-394	GAACGC	3e-10 (gb|EU088394)		AC027369.8 (LINE-1)	$2e^{-85}$
**71**	FM207228	1-277	250-386	TATATATTACACCAACCCCAA TCGAACC	1e-12 (gb|U07846)	5	AC106787.2 (Not determinate)	$6e^{-49}$
**71**	FM207229	1-274	262-488	CCCCAATCG AACC	5e-12 (gb|U07846)	8	AC018608.9 (LINE-1)	$5e^{-94}$
**71**	FM207230	34-323	1-56	ACGGC CCCTCCCAAA	1e-11 (gb|EU088401)	17	AC027793.9 (LINE-1)	$4e^{-04}$
**71**	FM207231	266-552	1-283	ACGCTGACC CCCCCTCCC	2e-10	4	AC110079.3 (LINE-1)	$2e^{-137}$

					(gb|EU088393)			
**75**	FM207232	55-119/242-356/456-570	1-54/116-246/345-461	AACC/ TACACC /CCCTCCCAAGAC	9e-54 (gb|AF399842)	21	CR381572.5 (LINE-1)	$3e^{-55}$
**75**	FM207233	111-121/325-355/629-742/842-948	1-118/128-332/335-635/732-849	TACACCAA/ AACC CCAATCGAACC/ CCTCCCAAGAC	1e-48 (gb|AF399842)	8	AC023533.6 (LINE-1)	$6e^{-104}$
**75**	FM207234	1-21/74-191/244-361	16-88/186-258/356-423	GGTGTA/ TTAGTCTTGGGAGGG	3e-58 (gb|AF399842)	X	AL732374.14 (LINE-1)	$2e^{-23}$
**75**	FM207235	1-137	122-611	AACCCCAATCGAACC	4e-21 (gb|AY490903)	14	AL137164.3 (LINE-1)	0.0
**75**	FM207236	193-484	1-295	GACCGCCCCTCCC	2e-22 (gb|AY490898)	1	AL513209.16 (LINE-1)	$5e^{-82}$
**75**	FM207237	642-926	1-653	TGAAGGCCCCTC	1e-09 (gb|EU088399)	4	AC079301.6 (LINE-1)	0.0
**81**	FM207238	254-371/681-798/1135-1252	1-260/357-687/784-1140	TACACCA/ CCCTCCC AAGACTAA	9e-39 (gb|AF399842)	X	AL732374.14 (LINE-1)	$4e^{-57}$
**81**	FM207239	22-139/405-522/575-691	1-27/125-410/508-580/687-1441	TACACC/ CCCTCC CAAGACTAA	6e-61 (gb|AF399842)	X	AL732374.14 (LINE-1)	$1e^{-58}$
**81**	FM207240	1-414	403-783	CCCTCCCA AGAC	5e-33 (gb|U07845)	X	AL732374.14 (LINE-1)	$6e^{-52}$
**81**	FM207241	1-603	600-755	AACC	3e-42 (gb|AF399842)	X	AL732374.14 (LINE-1)	$1e^{-32}$
**81**	FM207242	1-263/371-402	252-370/384-531	CCCAATCGAACC/ CACCAACCCCAATCGAACC	8e-10 (gb|EU088400)	7<br>12	AC012596.4 (LINE-1)<br>AC084364.20 (LINE-2)	$1e^{-45}$<br>$3e^{-41}$
**81**	FM207243	1-324/1261-	309-1275	CAACCCCAATCGAACC/	7e-22	5<br>12	AC108104.2 (LINE-1)	0.0

		1333		TTCGATTGGGGTTGG	(gb|EU088393)		AC084364.20 (Alu)	$7e^{-44}$
**81**	FM207244	414-733	1-422	GAACCCCCC	7e-12 (gb|EU088391)	5	AC091858.2 (Not determinate)	0.0
**85**	FM207245	1-380	366-671	CCCTCCCAAGACTAA	8e-43 (gb|AF399842)	X	AL732374.14 (LINE-1)	$5e^{-59}$
**85**	FM207246	1-378/475-484/517-627/703-803	364-481/483-523/620-708	CCCTCCCAAGACTAA/ TGAGTACACCA/ CCCAATCGAACC/ CTCTCCCA	1e-54 (gb|AF399842)	X	AL732374.14 (LINE-1)	$9e^{-44}$
**85**	FM207247	1-286/727-911	287-726	Non identified	3e-18 (gb|AY490898)	4	AC129664.7 (LINE-1)	0.0
**85**	FM207248	1-73	71-247	GGGAGGGGGG GTC	1e-15 (gb|EU088396)		AC019278.6 (LINE-1)	1e-^{74}
**85**	FM207249	177-258	1-189	GACGCCCCCTCCC	7e-13 (gb|EU088396)	2	AC007179.3 (LINE-1)	$8e^{-70}$
**85**	FM207250	1-82	70-642	GGGAGGGGCCGTC	8e-11 (gb|EU088396)	20	AL138805.8 (CYP24A1 gene)	0.0
**85**	FM207251	1-387/447-557/589-609/803-823	379-451/551-597/606-809/820-924	CCCTCCCAA/TACACC TACACCAAC/ AACC	2e-57 (gb|AF399842)	X	AL732374.14 (LINE-1)	$7e^{-78}$
**85**	FM207252	540-637	1-542	GA	2e-06 (gb|EU088391)	Y	AC010682.3 (LINE-1)	0.0
**85**	FM207253	159-502	1-175	GA CCCCCCCTCC CAAAA	7e-36 (gb|EU088399)	14	AL049776.3 (LINE-1)	$1e^{-71}$
