# Supplementary material for: Inheritance of DNA Transferred from American Trypanosomes to Human Hosts
Source: PLoS One. 2010 Feb 12;5(2):e9181. doi: 10.1371/journal.pone.0009181 (PMC2820539; doi:10.1371/journal.pone.0009181)
Supplement: Table S4 — Validation of the tpTAIL-PCR using different experimental protocols. (0.03 MB PDF) [file pone.0009181.s010.pdf]

Table S4. Validation of the *tp*TAIL-PCR using different experimental protocols*

Patient**	Cell source	Technique	Primers found	Clone	Amplicon sequence (nts)	kDNA/ host DNA (e-value)
C1	Somatic	*pt*TAIL-PCR	67rev/L1-2	5	kDNA (333)	emb|AJ747981.1| Trypanosoma cruzi kinetoplast minicircle, Expect = 3e-21
C1	Somatic	*pt*TAIL-PCR	67rev/L1-5	39	kDNA (64)	emb|AJ747986.1| Trypanosoma cruzi kinetoplast minicircle, Expect = 1e-21
C1	Somatic	*pt*TAIL-PCR	67rev/L1-2	79	kDNA (286)	gb|EF128032.1| Homo sapiens clone P2-13 trypanosome minicircle DNA integration region, Expect = 7e-29
C1	Somatic	*pt*TAIL-PCR	67rev/L1-2	89	kDNA (286)	gb|AF242562.1| Trypanosoma cruzi clone FGz 8-3 kinetoplast minicircle sequence, Expect = 2e-10
C1	Somatic	*pt*TAIL-PCR	67rev/L1-2	104	kDNA (502)	gb|AY584193.1| Homo sapiens clone G10 Trypanosoma cruzi infected macrophage p15 gene disrupted by LINE-1 element and Trypanosoma cruzi minicircle kinetoplast sequence, Expect = 1e-135
C1	Somatic	*pt*TAIL-PCR	67rev/L1-2	106	kDNA (224)	gb|EF128032.1| Homo sapiens clone P2-13 trypanosome minicircle DNA integration region, Expect = 6e-08
C2	Somatic	*pt*TAIL-PCR	67rev/L1-2	114	kDNA (492)	gb|AF399842.1|AF399842 Trypanosoma cruzi clone 5 integrated kinetoplast, Expect = 1e-46
C2	Somatic	*pt*TAIL-PCR	67rev/L1-5	123	kDNA (101)	gb|AY584193.1| Homo sapiens clone G10 Trypanosoma cruzi infected macrophage p15 gene disrupted by LINE-1 element and Trypanosoma cruzi minicircle, Expect = 7e-

						27
C2	Somatic	*pt*TAIL-PCR	67rev/L1-5	140	kDNA (388)	gb|M18814.1|TRBKPMCAA T.cruzi kinetoplast minicircle DNA, clone KY-13, Expect = 1e-45
C2	Somatic	*pt*TAIL-PCR	67rev/L1-2	156	kDNA (291)	gb|AF241157.1| Trypanosoma cruzi clone FGz 71 kinetoplast minicircle DNA, Expect = 3e-39
C2	Somatic	*pt*TAIL-PCR	67rev/L1-2	162	kDNA (263)	gb|AF242563.1| Trypanosoma cruzi clone FGz 8-4 kinetoplast minicircle sequence, Expect = 4e-08<br>gb|AC079399.5| Homo sapiens BAC clone RP11-414P19 from 4, Expect = 0.0
C2	Somatic	*pt*TAIL-PCR	67rev/67rev	172	kDNA (454)	emb|X04680.1|MITCMNC Trypanosoma cruzi minicircle DNA, Expect = 2e-42
C2	Somatic	*pt*TAIL-PCR	67rev/L1-5	176	kDNA (364)	gb|M19188.1|TRBKPMCO T.cruzi kinetoplast minicircle DNA, clone y01 cst 4<br>Expect = 2e-49
C2	Somatic	*pt*TAIL-PCR	67rev/L1-5	199	kDNA (341)	gb|M19187.1|TRBKPMCN T.cruzi kinetoplast minicircle DNA, clone y01 cst 3, Expect = 8e-48
C2	Somatic	*pt*TAIL-PCR	67rev/L1-2	250	kDNA (130)	gb|AF399842.1|AF399842 Trypanosoma cruzi clone 5 integrated kinetoplast minicircle DNA, Expect = 3e-10
C2	Somatic	*pt*TAIL-PCR	67rev/L1-2	256	kDNA (100)	gb|M19185.1|TRBKPMCL T.cruzi kinetoplast minicircle DNA, Expect = 3e-25
C2	Somatic	*pt*TAIL-PCR	67rev/L1-2	264	kDNA (418)	emb|X04680.1|MITCMNC Trypanosoma cruzi minicircle DNA, Expect = 1e-34
C2	Somatic	*pt*TAIL-PCR	67rev/67rev	265	kDNA (232)	gb|U07846.1|TCU07846 Trypanosoma cruzi Y kinetoplast minicircle sequence, Expect = 4e-17
C2	Somatic	*pt*TAIL-	67rev/L1-2	266	kDNA (548)	gb|M19187.1|TRBKPMCN T.cruzi kinetoplast

		PCR				minicircle DNA, clone y01 cst 3, 2e-43
C2	Somatic	*pt*TAIL-PCR	67rev/67rev	274	kDNA (625)	gb|M18814.1|TRBKPMCAA T.cruzi kinetoplast minicircle DNA, Expect = 5e-21
C2	Somatic	*pt*TAIL-PCR	67rev/L1-5	275	kDNA (388)	emb|AJ747993.1| Trypanosoma cruzi kinetoplast minicircle, lineage IIb, Expect = 3e-104
C2	Somatic	*pt*TAIL-PCR	67rev/67rev	280	kDNA (1342)	emb|AJ747993.1| Trypanosoma cruzi kinetoplast minicircle, lineage IIb, Expect = 4e-108
C2	Somatic	*pt*TAIL-PCR	67rev/L1-5	293	kDNA (694)	gb|M18814.1|TRBKPMCAA T.cruzi kinetoplast minicircle DNA, Expect = 1e-54
16	Somatic	PCR	67rev/67rev	73	kDNA (1- 295)<br>Host DNA (278-914)	gb|U07846.1|TCU07846 Trypanosoma cruzi Y kinetoplast minicircle sequence, Expect = 6e-08<br>gb|AC007741.4| Homo sapiens BAC clone RP11-340F16 from 2, Expect = 0.0
16	Somatic	PCR	67rev/L1-5	85	kDNA (360)	gb|M19188.1|TRBKPMCO T.cruzi kinetoplast minicircle DNA, Expect = 2e-42
16	Somatic	PCR	67rev/L1-5	88	kDNA (1-387)<br>Host DNA (373-465)	gb|M19188.1|TRBKPMCO T.cruzi kinetoplast minicircle DNA, Expect = 9e-48<br>emb|AL732374.14| Human DNA sequence from clone RP13-444K19 on chromosome X, Expect = 8e-36
16	Somatic	PCR	67rev/67rev	92	kDNA (1-295/488-773<br>Host DNA (284-499)	gb|U07846.1|TCU07846 Trypanosoma cruzi Y kinetoplast minicircle sequence, Expect = 9e-11<br>gb|AC084364.20| Homo sapiens 12q BAC RP11-626I20, Expect =4e-42 / gb|AC012596.4| Homo sapiens BAC clone CTD-2523K17 from 7, Expect = 4e-48
27	Somatic	PCR	67rev/L1-5	32	kDNA (360)	gb|M19188.1|TRBKPMCO T.cruzi kinetoplast minicircle DNA, Expect = 4e-45
50	Somatic	PCR	67rev/67rev	115	kDNA (328)	gb|AY289115.1| Trypanosoma cruzi clone 8-13 integrated kinetoplast minicircle,

						Expect = 9e-08
50	Somatic	PCR	67rev/L1-5	120	kDNA (360)	gb|M19188.1|TRBKPMCO T.cruzi kinetoplast minicircle DNA, Expect = 6e-49
50	Somatic	PCR	L1-5/L1-5	131	Host DNA (1589)	emb|AL162420.13| Human DNA sequence from clone RP11-380P16 on chromosome 9, Contains the IFNA17 gene for interferon, Expect = 0.0
55	Somatic	PCR	67rev/67rev	156	kDNA (328)	gb|AY289115.1| Trypanosoma cruzi clone 8-13 integrated kinetoplast minicircle, Expect = 9e-08
55	Somatic	PCR	67rev/L1-5	161	kDNA (380)	emb|AJ748036.1| Trypanosoma cruzi kinetoplast minicircle, Expect = 3e-135
55	Somatic	PCR	67rev/L1-5	168	kDNA (360)	gb|M19188.1|TRBKPMCO T.cruzi kinetoplast minicircle DNA, Expect = 6e-49
56	Somatic	PCR	67rev/67rev	173	kDNA (328)	gb|AY289115.1| Trypanosoma cruzi clone 8-13 integrated kinetoplast minicircle, Expect = 9e-08
59	Somatic	PCR	67rev/L1-5	188	kDNA (380)	emb|AJ748036.1| Trypanosoma cruzi kinetoplast minicircle, Expect = 3e-135
59	Somatic	PCR	67rev/L1-5	198	Host DNA (165)	emb|AL732374.14| Human DNA sequence from clone RP13-444K19 on chromosome X, Expect = 1e-46
6	Germline	PCR	67rev/L1-5	E-247	kDNA (104)	emb|X04680.1|MITCMNC Trypanosoma cruzi minicircle DNA,Expect =6e-22
6	Germline	PCR	L1-5/L1-5	E-249	Host DNA (1610)	emb|AL162420.13| Human DNA sequence from clone RP11-380P16 on chromosome 9 Contains the IFNA17 gene for interferon, Expect= 0.0
6	Germline	PCR	67rev/L1-5	E-250	kDNA (358)	gb|M19185.1|TRBKPMCL T.cruzi kinetoplast minicircle DNA, clone y01 cst 1,Expect= 1e-45
25	Germline	PCR	67rev/67rev	E-254	kDNA (232)	emb|AJ747996.1| Trypanosoma cruzi kinetoplast minicircle, lineage IIb, strain Tu18 cl2, isolate 02mp37, Expect = 4e-17
25	Germline	PCR	67rev/L1-5	E-256	kDNA (369)	gb|M19188.1|TRBKPMCO T.cruzi kinetoplast minicircle DNA, clone y01 cst 4,Expect = 1e-39

25	Germline	PCR	67rev/L1-5	E-258	kDNA (371)	gb|M18814.1|TRBKPMCAA T.cruzi kinetoplast minicircle DNA, clone KY-13, Expect = 9e-167
34	Germline	PCR	67rev/L1-5	E-259	kDNA (81)	emb|X04680.1|MITCMNC Trypanosoma cruzi minicircle DNA, Expect = 2e-21
34	Germline	PCR	L1-5/L1-5	E-261	Host DNA (1610)	emb|AL162420.13| Human DNA sequence from clone RP11-380P16 on chromosome 9 Contains the IFNA17 gene for interferon, Expect= 0.0
71	Germline	PCR	67rev/L1-5	E-262	kDNA (100)	emb|X04680.1|MITCMNC Trypanosoma cruzi minicircle DNA, Expect = 5e-23
71	Germline	PCR	67rev/L1-5	E-263	kDNA (371)	gb|M18814.1|TRBKPMCAA T.cruzi kinetoplast minicircle DNA, clone KY-13, Expect = 1e-164
71	Germline	PCR	67rev/L1-5	E-264	kDNA (1-380) Host DNA (381-530)	emb|AJ748036.1| Trypanosoma cruzi kinetoplast minicircle, lineage IIe, strain CL Brener, isolate JP726,Expect = 4e-135 emb|AL732374.14| Human DNA sequence from clone RP13-444K19 on chromosome X Contains a mitochondrial ribosomal protein S18C (MRPS18C) pseudogene,the 3' end of the PHF8 gene for PHD finger protein 8 and a CpG island, complete sequence, Expect = 1e-52
71	Germline	PCR	67rev/L1-5	E-267	kDNA (388 bp)	gb|M18814.1|TRBKPMCAA T.cruzi kinetoplast minicircle DNA, clone KY-13, Expect = 1e-45
71	Germline	PCR	67rev/L1-5	E-269	kDNA (85 bp)	emb|X04680.1|MITCMNC Trypanosoma cruzi minicircle DNA, Expect = 1e-22
71	Germline	PCR	67rev/L1-5	E-272	Host DNA (1-93) kDNA (94-141)	gb|U07846.1|TCU07846 Trypanosoma cruzi Y kinetoplast minicircle sequence, clone M369r, orf, complete cds,Expect = 2e-15 emb|AL732374.14| D Human DNA sequence from clone RP13-444K19 on chromosome X Contains a mitochondrial ribosomal protein S18C (MRPS18C) pseudogene,the 3' end of the PHF8 gene for PHD finger protein 8 and a CpG island,Expect = 2e-36

81	Germline	PCR	67rev/L1-5	E-279	kDNA (1-263) Host DNA (264-326)	gb|AC233981.2| Homo sapiens BAC clone RP11-620C18 from chromosome x,Expect = 4e-25
81	Germline	PCR	67rev/L1-5	E-280	kDNA (1-387) Host DNA (388-465)	gb|M19188.1|TRBKPMCO T.cruzi kinetoplast minicircle DNA, clone y01 cst 4,Expect =9e-48<br>emb|AL732374.14| Human DNA sequence from clone RP13-444K19 on chromosome X Contains a mitochondrial ribosomal protein S18C (MRPS18C) pseudogene,the 3' end of the PHF8 gene for PHD finger protein 8 and a CpG island, Expect =3e-34
81	Germline	PCR	67rev/L1-5	E-282	kDNA (387 bp)	gb|M19188.1|TRBKPMCO T.cruzi kinetoplast minicircle DNA, clone y01 cst 4,Expect = 4e-45

* A mix of wild-type *T. cruzi* kDNA with DNA from two control individuals (C1 and C2) born in Europe were used as templates for the *tp*TAIL-PCR amplifications. This procedure aimed at the specificity of the *tp*TAIL-PCR used to show the kDNA integrations in the population belonging to five studied families.

** The blood mononuclear cells or the germline cells from the studied families patients showing the kDNA positive exams were used, and the results obtained with the traditional PCR technique can be compared with those obtained with the modified *tp*TAIL-PCR used in the study.
