# Supplementary material for: Inheritance of DNA Transferred from American Trypanosomes to Human Hosts
Source: PLoS One. 2010 Feb 12;5(2):e9181. doi: 10.1371/journal.pone.0009181 (PMC2820539; doi:10.1371/journal.pone.0009181)
Supplement: Table S5 — Lateral transfer of Trypanosoma cruzi kDNA minicircle sequences provoking gene knock-out into the human genome. (0.01 MB PDF) [file pone.0009181.s011.pdf]

Table S5. Lateral Transfer of *Trypanosoma cruzi* kDNA Minicircle Sequences Provoking Gene Knock-out into the Human Genome

Patient	Gene	Function	GeneBank	*E-value*	Similarity
5	PARP-1	ADP-ribosyltransferase	emb|AL359742.15	$1.0e^{-12}$	LINE-1 (0.012)
5)	CNTNAP2	Adhesion molecules and receptors in the vertebrate nervous system	ref|NG_007092.2|	$4.0e^{-38}$	LINE-1 (8.0e^{-14}
15	OR1-17	Olfactory receptor	tpg|BK004196.1	$7.0e^{-17}$	--
19	ADAM-23	Implicated in cell-cell and cell-matrix interactions including fertilization, muscle development. and neurogenesis. It is highly expressed in the brain where it may function as an integrin ligand.	gb|AC009225.4|	0	--
26	CLIC-5	chloride intracellular channel	emb|AL355522.11|	$2.0e^{-137}$	--
28	TRIM69	Member of the RING-B-box-coiled-coil (RBCC) family it encodes a protein with an N-terminal RING finger motif. a PRY domain and a C-terminal SPRY domain.	dbj|AK292252.1|	$2.0e^{-45}$	--
30	OR1-17	Olfactory receptor	tpg|BK004196.1	$2.0e^{-16}$	--
36	Theta-14-3-3	Mediate signal transduction by binding to phosphoserine-containing proteins. Upregulated in patients with amyotrophic lateral sclerosis.	gb|BC001197.1|	0	Transposon (0)
44)	PHD finger protein 15	Several PHD fingers acting as binding modules of methylated histone H3.	ref|NM_015288.4	$3.0e^{-93}$	--
56	OR1-17	Olfactory receptor	tpg|BK004196.1	$2.0e^{-20}$	--
63	citb-109	--	gb|AC005871.3|AC005871	$2.0e^{-136}$	--
69	Haplotype HLA	Major histocompatibility complex class I	gb|DQ249181.1|	0	LINE-1 (1.0e^{-102}
74	OR1-17	Olfactory receptor	tpg|BK004196.1	$2.0e^{-20}$	--

75	Tyrosine -phosphatase	Protein tyrosine-phosphatase. receptor type	emb|AL590397.7|	$5.0e^{-132}$	--
81	OR1-17	Olfactory receptor	tpg|BK004196.1	$6.0e^{-14}$	--
82	OR1-17	Olfactory receptor	tpg|BK004196.1	$3.0e^{-9}$	--
82	OR1-17	Olfactory receptor	tpg|BK004196.1	$1.0e^{-18}$	--
